# Recent progress in small-molecule fluorescent probes for the detection of superoxide anion, nitric oxide, and peroxynitrite anion in biological systems

**DOI:** 10.1039/d4sc06722c

**Published:** 2024-11-20

**Authors:** Yongqing Zhou, Xuan Kuang, Xiaofeng Yang, Juan Li, Xianzhe Wei, Won Jun Jang, Shan-Shan Zhang, Mei Yan, Juyoung Yoon

**Affiliations:** a School of Chemistry and Chemical Engineering, University of Jinan Jinan 250022 People's Republic of China chm_yanm@126.com; b Shandong Provincial Key Laboratory of Fluorine Chemistry and Chemical Materials, University of Jinan Jinan 250022 People's Republic of China; c College of Chemistry, Chemical Engineering and Materials Science, Collaborative Innovation Center of Functionalized Probes for Chemical Imaging in Universities of Shandong, Key Laboratory of Molecular and Nano Probes, Ministry of Education, Shandong Normal University Jinan 250014 People's Republic of China sszhangchem@126.com; d Key Laboratory of Optic-Electric Sensing and Analytical Chemistry for Life Science, MOE, College of Chemistry and Molecular Engineering, Qingdao University of Science and Technology Qingdao 266042 People's Republic of China; e Department of Chemistry and Nanoscience, Ewha Womans University Seoul 03760 Korea jyoon@ewha.ac.kr

## Abstract

Superoxide anion (O_2_˙^−^), nitric oxide (NO), and peroxynitrite anion (ONOO^−^) play essential roles in physiological and pathological processes, which are related to various symptoms and diseases. There is a growing need to develop reliable techniques for effectively monitoring the changes in these three reactive species across different molecular events. Currently, small-molecule fluorescent probes have been demonstrated to be reliable imaging tools for the optical detection and biological analysis of reactive species in biological systems due to their high spatiotemporal resolution and *in situ* capabilities. In consideration of the distinct features of these three reactive species, abundant fluorescent probes have been developed to meet various requirements. In this context, we systematically summarized the latest progress (2020–2023) in organic fluorescent probes for monitoring O_2_˙^−^, NO, and ONOO^−^ in living systems. Furthermore, the working principles and biological applications of representative fluorescent probes were illustrated. Moreover, we highlighted the current challenges and future trends of fluorescent probes, offering general insights into future research.

## Abstract 2

In this review, we summarized the latest progress (2020–2023) in small-molecule fluorescent probes for monitoring superoxide anion (O_2_˙^−^), nitric oxide (NO), and peroxynitrite anion (ONOO^−^) in biological systems.

## Graphical abstract

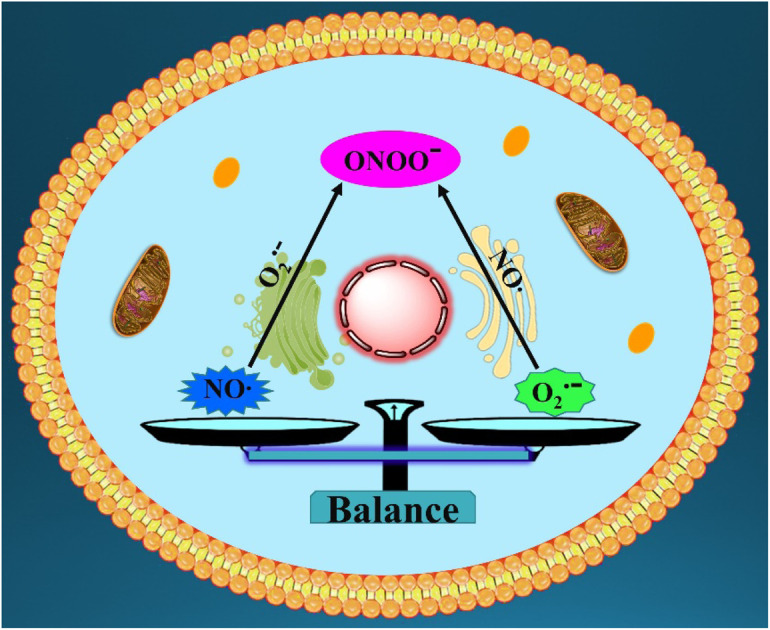

## 1 Introduction

**Fig. 1:**
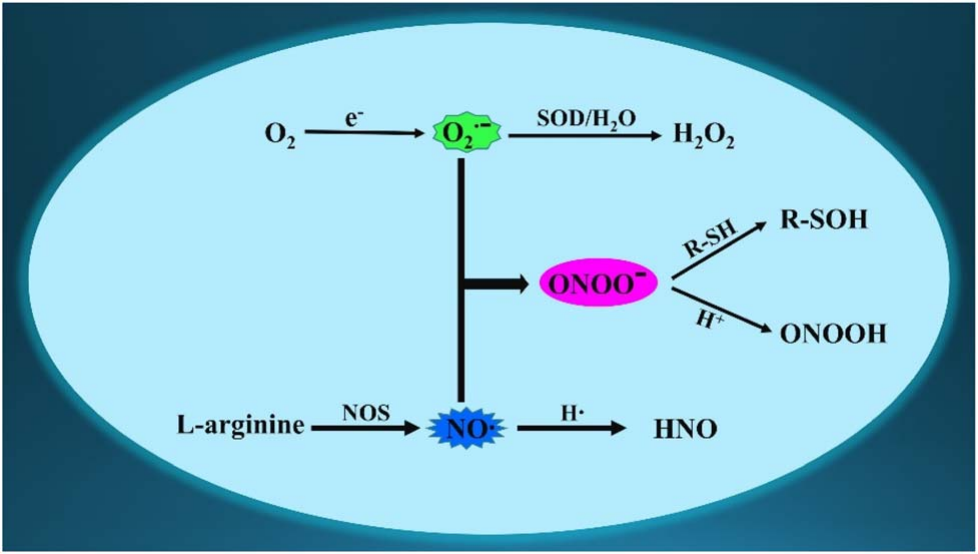
Schematic for the generation and conversion of O_2_˙^−^, NO, and ONOO^−^ in living cells.

Endogenous reactive species are crucial for maintaining metabolism and redox balance.^1,2^ Among the many bioactive molecules, the superoxide anion (O_2_˙^−^), the one-electron reduction product of oxygen, is the first produced intracellular reactive oxygen species (ROS).^3^ Moreover, nitric oxide (NO or NO˙), a crucial gas molecule, is primarily generated during the biosynthesis of l-citrulline from l-arginine.^4^ Peroxynitrite anion (ONOO^−^), a potent oxidizing and nitrifying reactive nitrogen species (RNS), is formed by the diffusion-limited recombination of O_2_˙^−^ and NO.^5^ Under physiological conditions, the levels of O_2_˙^−^ or NO directly determine the concentrations of ONOO^−^. However, if the concentration of any reactive species changes, it disrupts the physiological balance among these three bioactive species, leading to adverse symptoms. Consequently, there must be some interactions and relationships between the three reactive species (O_2_˙^−^, NO and ONOO^−^) in biological systems (Fig. 1).^6^ As we all know, these three reactive species are recognized as potential intracellular messengers under physiological concentrations, which regulate several signaling pathways. For example, NO serves as a ubiquitous signaling molecule in cellular activities.^7^ However, the upregulated expression of the three reactive species disrupts the structures of certain biomolecules, including enzymes and nucleic acids, causing intracellular oxidative stress.^8^ Moreover, several reports have indicated that the high expression of the three reactive species is associated with pathological processes and diseases, such as liver damage, epilepsy, Alzheimer's disease (AD), and cancer.^9–11^ Hence, developing precise identification methods for O_2_˙^−^, NO, and ONOO^−^ are crucial for exploring their generation, distribution, elusive functions, underlying mechanisms, interrelationship, and synergistic regulations in biological systems.

In view of their complex physiological characteristics, such as low concentrations, short-lived existence, and wide distributions,^12^ directly visualizing changes in these three bioactive molecules across various physiological, pathological, and pharmacological processes remain a challenge. Compared with other detection methods, fluorescence imaging technology offers several advantages in precision detection and analysis, including high spatiotemporal resolution, noninvasiveness and *in situ* operation.^13–33^ Organic fluorescent probes have successfully served as practical instruments for the real-time monitoring of variations in reactive species. Currently, numerous organic fluorescent probes have been skillfully developed for detecting fluctuations in the three reactive species (O_2_˙^−^, NO, and ONOO^−^), revealing diverse biological functions in living systems.

This comprehensive review discusses recent advances in organic fluorescence probes for monitoring O_2_˙^−^, NO, and ONOO^−^ in biological systems. The design strategies of O_2_˙^−^, NO, and ONOO^−^-responsive fluorescent probes have been elaborately discussed, focusing on fluorophores, ligands, and recognition groups. Additionally, to enhance reader's comprehension of this compelling field, we have systematically classified the content into two sections: single-analyte and two-analyte detection. The first section is further divided into three subsections corresponding to each species (O_2_˙^−^, NO, and ONOO^−^). Each subsection prominently emphasizes the design strategies, working principles, biological applications, and molecular mechanisms of significant examples. Moreover, with the aid of these advanced fluorescent probes, the production, conversion, functions, and adverse effects of the three reactive species have been completely imaged and analyzed under various physiological and pathological conditions. We also address the current challenges and future opportunities in the development of molecular probes for detecting O_2_˙^−^, NO, and ONOO^−^ in biological systems.

## 2 Small-molecule fluorescent probes for single-analyte analysis

### 2.1 Monitoring of O_2_˙^−^

O_2_˙^−^ is a critical and widely distributed ROS in the cytoplasmic matrix, mitochondria, endoplasmic reticulum, and so on.^34,35^ It is generated when electrons leak from the electron transport chain to oxygen. Moreover, O_2_˙^−^ is also the “primary” ROS, which can further convert into other ROS/RNS. For example, O_2_˙^−^ react with NO to generate ONOO^−^.^36^ To date, O_2_˙^−^ has been considered as a potential messenger for regulating cellular signaling networks under physiological conditions.^37^ Moreover, the normal concentration of O_2_˙^−^ is crucial for maintaining intracellular oxidative balance. However, the excessive accumulation of O_2_˙^−^ and other ROS/RNS disrupt intracellular homeostasis, leading to oxidative damage to proteins and lipids. This process can further impair organelle and cytoskeleton structures, ultimately bringing about various pathological symptoms and diseases, including ischemia-reperfusion injury, liver injury, and depression. To accurately track O_2_˙^−^ fluctuations during various molecular events in biological systems, numerous organic fluorescent probes based on different recognition reactions have been developed, including the oxidation of the pyrocatechol unit and the nucleophilic substitution reaction of diphenylphosphinyl and trifluoromethanesulfonate groups (Table 1).

**Table 1:** Some detection parameters and application of O_2_˙^−^-responsive small-molecule fluorescent probes are summarized

Probe name	Excitation wavelengths (nm)	Emission wavelengths (nm)	Targeting region	Detection limits	Biological application	References
TCP	370	495	Peroxisome	21.5 nM	In cell and mice with depression	38
CPR-SK	400	580/470	Cytoplasm	2.35 nM	In ischemia reperfusion injury cells	39
DPC	370	480	Endoplasmic reticulum	0.18 μM	In cells and hepatic ischemia-reperfusion injury mice	40
PA-CA	370	490	Cytoplasm	705.9 nM	In cells and the brains of living mice	41
HT-CA	370	490	Cytoplasm	496.9 nM	In cells and the brains of living mice	
Per*qdOH*	700/490	750/540	Mitochondria	—	In cells and the lesions of primary syphilis model	42
RDX	580	638	Cytoplasm	2.09 μM	In cells and diabetic mice	43
MB-SO	590	690	Cytoplasm	14 nM	In cells and in mice	44
BODIPY-T	480	530	Cytoplasm	0.062 μM	In cells	45
DMPS-O	418	635	Mitochondria	2.22 × 10^−8^ M	In cells	46
NR1	590	650	Cytoplasm	0.34 μM	In cells	47
MAP-O_2_˙^−^	670	721	—	262.8 nM	In drug-induced hepatotoxicity mice	48
CT-CF_3_	500	670	Cytoplasm	0.079 μM	In cells and adolescent mice	49
TCF-OTf	560	606	—	—	In a model antibiotic with *S. aureus*,*P. aeruginosa*,*E. coli*, and *E. faecalis*	50
Gol-Cou-O_2_˙^−^	405	460	Golgi apparatus	3.9 × 10^−7^ M	In cells and zebrafish	51
DLS4	600	660	Cytoplasm	7.3 nM	In cells	52
TPER-O_2_˙^−^	450	554	Endoplasmic reticulum	3.3 × 10^−7^ M	In cells and APAP-induced mouse liver slices	53
ER-Tf	405	462	Endoplasmic reticulum	65 nM	In cells	54
ER-Rs	500	558	Endoplasmic reticulum	012 μM	In cells, zebrafish and a fresh liver tissue from a mouse	55
Lyso-MHC	450	556	Lysosome	0.047 nM	In cells, zebrafish and lung tissue slices of mice	56
IFP-O_2_	500	645	Mitochondria	10 nM	In cells	57
NAP-SCM	390	454	Cytoplasm	78 nM	In cells and zebrafish	58
XM-TBS	690	821	Cytoplasm	33.8 nM	In cells and DILI mice	59

#### 2.1.1 Fluorescent probes based on the pyrocatechol unit

**Fig. 2:**
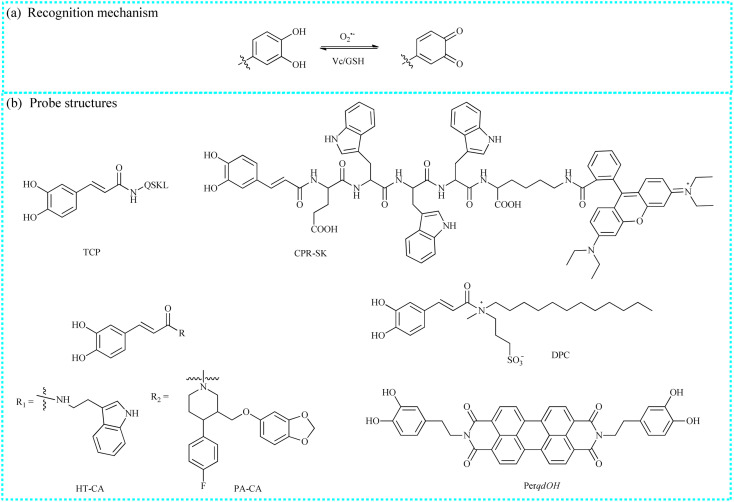
(a) The recognition mechanism of the pyrocatechol unit and O_2_˙^−^. (b) The structures of typical fluorescent probes.

**Fig. 3:**
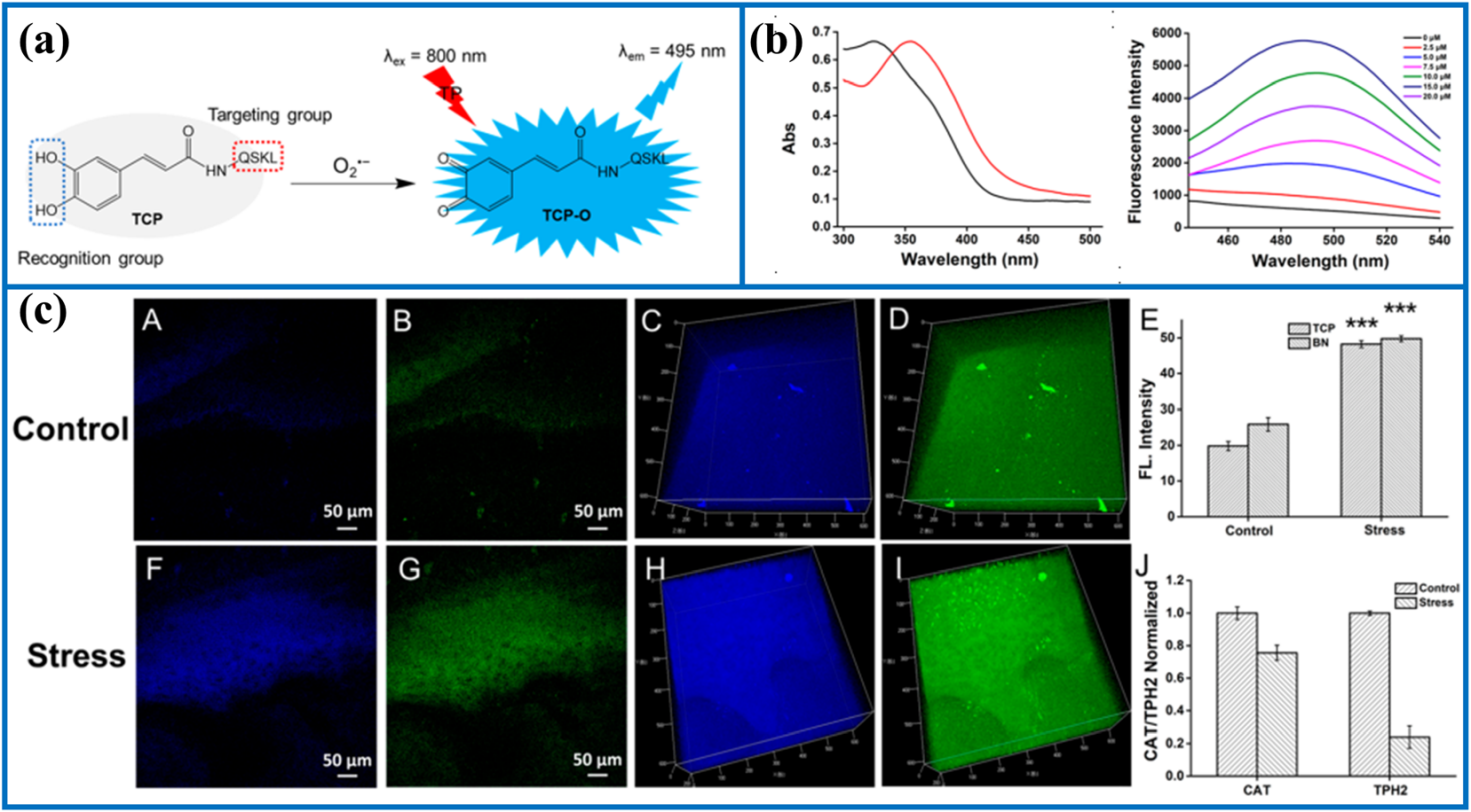
(a) The recognition mechanism between probe TCP and O_2_˙^−^. (b) The absorption and fluorescence spectra of TCP before and after activation by O_2_˙^−^. (c) Two-photon fluorescence imaging in the brain of the control and stressed mouse. This figure has been reproduced from ref. 38 with permission from American Chemical Society, copyright 2020.

Tang's group skillfully synthesized a novel two-photon fluorescent probe (TCP) to monitor peroxisomal O_2_˙^−^ concentrations in real-time and *in situ*.^38^ The sensing system consisted of two segments: small peptides (QSKL) and caffeic acid (Fig. 2). Among them, the SKL peptides served as the peroxisomal targeting group. Before reacting with O_2_˙^−^, TCP was nonfluorescent. Upon reacting with O_2_˙^−^, the pyrocatechol unit group (electron-donating group) in the probe structure rapidly converted into a carbonyl moiety (electron-withdrawing group), resulting in illuminous blue fluorescence (Fig. 3). With the help of two-photon fluorescence imaging and proteomic analysis, the authors clearly observed that high concentrations of peroxisomal O_2_˙^−^ caused the inactivation of catalase (CAT), resulting in increased levels of hydrogen peroxide (H_2_O_2_) under oxidative stress. Moreover, the overexpression of H_2_O_2_ oxidized tryptophan hydroxylase-2 (TPH2), resulted in the dysregulation of the 5-hydroxytryptamine, and ultimately accelerated the onset of depression. In addition, the authors also clearly identified the sites of oxidative modification on CAT by peroxisomal O_2_˙^−^ and on TPH2 by superfluous intracellular H_2_O_2_. In summary, this study fully elucidated the signaling pathway mediated by peroxisomal O_2_˙^−^ in the initiation and progression of depression and contributed to identifying several emerging key targets for depression therapy.

To achieve the precise detection of intracellular O_2_˙^−^ levels, this team introduced a new ratiometric fluorescent probe (CPR-SK) utilizing the fluorescence resonance energy transfer (FRET) mechanism.^39^ Caffeic acid, rhodamine B, and targeting peptides (EWWW) were selected to construct this molecular probe. CPR-SK was specifically activated by O_2_˙^−^ in the buffer solutions, emitting strong fluorescence. Moreover, rhodamine B served as the energy receptor since its absorption spectra partially overlapped with the blue emission spectra of the reaction product spectra of caffeic acid and O_2_˙^−^. CPR-SK is considered as a versatile tool for the real-time study of intracellular molecular events under oxidative stress, including O_2_˙^−^ fluctuations and Keap1 migration. The study results showed that CPR-SK could monitor the increase in the O_2_˙^−^ levels under oxidative stress and track the migration of Keap1 from the cytoplasm to the nucleus. Therefore, the findings indicated that the high spatiotemporal imaging method employed in this study could be used to monitor the dynamic changes of O_2_˙^−^ in intracellular redox homeostasis and elucidate the molecular mechanisms of oxidative stress-related diseases. Moreover, using this O_2_˙^−^ recognition unit, Tang's group successfully developed a series of small-molecule fluorescent tools (DPC, PA-CA and HT-CA) to visualize O_2_˙^−^ variations and reveal elusive molecular mechanisms.^40,41^

Liao's group developed a new organic fluorescent sensor (Per*qdOH*) by integrating a fluorophore (perylene) and a recognition group (dopamine). This sensor enabled dynamic and reversible detection of intracellular O_2_˙^−^ variations.^42^ Upon exposure to O_2_˙^−^, the pyrocatechol unit in Per*qdOH* was selectively oxidized to a carbonyl structure, resulting in a marked color change from green to red in the reaction solution. Moreover, the addition of glutathione (GSH) enhanced the emission signals at 540 nm and attenuated another emission at 750 nm in the fluorescent spectra. With the advantages of Per*qdOH*, such as near-infrared (NIR) emission, they successfully measured endogenous O_2_˙^−^ concentrations in a lesion of a primary syphilis model.

#### 2.1.2 Fluorescent probes based on the diphenylphosphinyl group

**Fig. 4:**
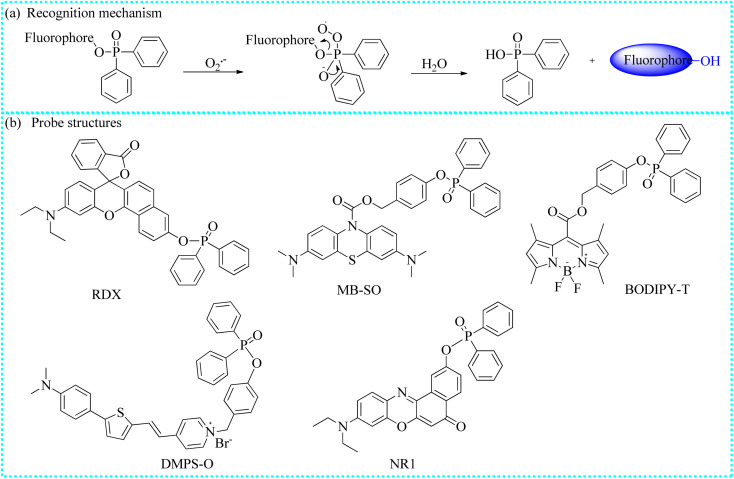
(a) The recognition mechanism between diphenylphosphinyl group and O_2_˙^−^. (b) The structures of some fluorescent probes.

Taking advantage of the strong nucleophilic nature of O_2_˙^−^, a novel NIR fluorescent probe (RDX) was developed by linking a Rho fluorophore and diphenylphosphinyl group (Fig. 4).^43^ The initial fluorescence signals of the control RDX were relatively weak owing to the caging of the OH group in the fluorophore by the triggering moiety. The diphenylphosphonate site in the RDX was selectively reacted with O_2_˙^−^, triggering the release of the Rho fluorophore in its open ring form, resulting in strong fluorescence emission. *In vitro* experimental results revealed that RDX exhibited greater sensitivity and selectivity towards O_2_˙^−^ than the other species. Moreover, employing one-photon and two-photon fluorescence imaging technologies, authors consecutively observed the increase in intracellular O_2_˙^−^ concentrations in response to various stimuli such as lipopolysaccharide (LPS) or 2-methoxyestradiol. Nevertheless, intracellular fluorescent signals were significantly suppressed following treatment with GSH or Trion. Notably, RDX was effectively utilized to track the variations of O_2_˙^−^ in diabetic mice. Compared to the control or treatment groups, diabetic mice exhibited significantly stronger fluorescence, suggesting that these diabetic mice generated a substantial amount of O_2_˙^−^.

Zhang's group knowledgeably proposed a new NIR fluorescent probe (MB-SO), which was used to monitor endogenous O_2_˙^−^ fluxes.^44^*In vitro* experiments showed a positive correlation between the fluorescence values of the reacting solutions and O_2_˙^−^ content. Additionally, MB-SO was used to visualize endogenous O_2_˙^−^ levels in the hippocampi of mouse brains with pentylenetetrazole-induced epilepsy. Meantime, these preliminary data revealed a positive correlation between high O_2_˙^−^ content in mouse brains and epileptogenesis. Herein, MB-SO could serve as a detection tool to assess O_2_˙^−^ levels and reveal the pathogenesis of epilepsy.

Peng's group designed a new turn-on fluorescent sensing probe (BODIPY-T) by modifying the BODIPY dye for the specific recognition of intracellular O_2_˙^−^ content.^45^ The developed probe exhibited excellent performance for *in vitro* O_2_˙^−^ detection, mainly including negligible interference from other species and a wide pH range. In addition, the endogenous O_2_˙^−^ changes in RAW264.7 cells were successfully realized by imaging through confocal microscopy, suggesting that BODIPY-T may be an efficient platform for explaining certain diseases and the associated molecular mechanisms.

To visualize O_2_˙^−^ fluxes in mitochondria, Hua's group elaborately developed a novel fluorescent probe (DMPS-O) incorporating *N*,*N*-dimethylaniline, pyridinium, and diphenylphosphinate groups.^46^ A significant Stokes shift (217 nm) was observed in the spectra before and after DMPS-O reacted with O_2_˙^−^. This phenomenon was beneficial to avoid biological background fluorescence and improve the imaging accuracy. Furthermore, confocal fluorescent imaging experiments demonstrated that cells stimulated by LPS exhibited stronger fluorescence. However, the intense fluorescence of these stimulated cells was dramatically suppressed after incubation with Tiron, suggesting that DMPS-O could detect O_2_˙^−^ concentrations. Ultimately, these experimental evidences fully proved that DMPS-O was suitable for *in situ* monitoring of the O_2_˙^−^ levels in biological environments.

The phosphate bond linking Nile red dye and diphenylphosphonate in an NIR fluorescent probe (NR1) was utilized for sensing intracellular variations in O_2_˙^−^ levels.^47^ Upon exposure to other interfering biospecies, NR1 highly selectively monitored O_2_˙^−^ in selectivity experiments. Furthermore, it showed a stable fluorescence response to O_2_˙^−^ in only two minutes. Moreover, a strong linear fitting curve between the emission intensities of the reacting solutions and probe concentrations was smoothly gained. Utilizing this NR1, the fluctuations in O_2_˙^−^ were successfully imaged in the cancer cell lines (4T1 and HeLa cells).

#### 2.1.3 Fluorescent probes based on trifluoromethanesulfonate ester

**Fig. 5:**
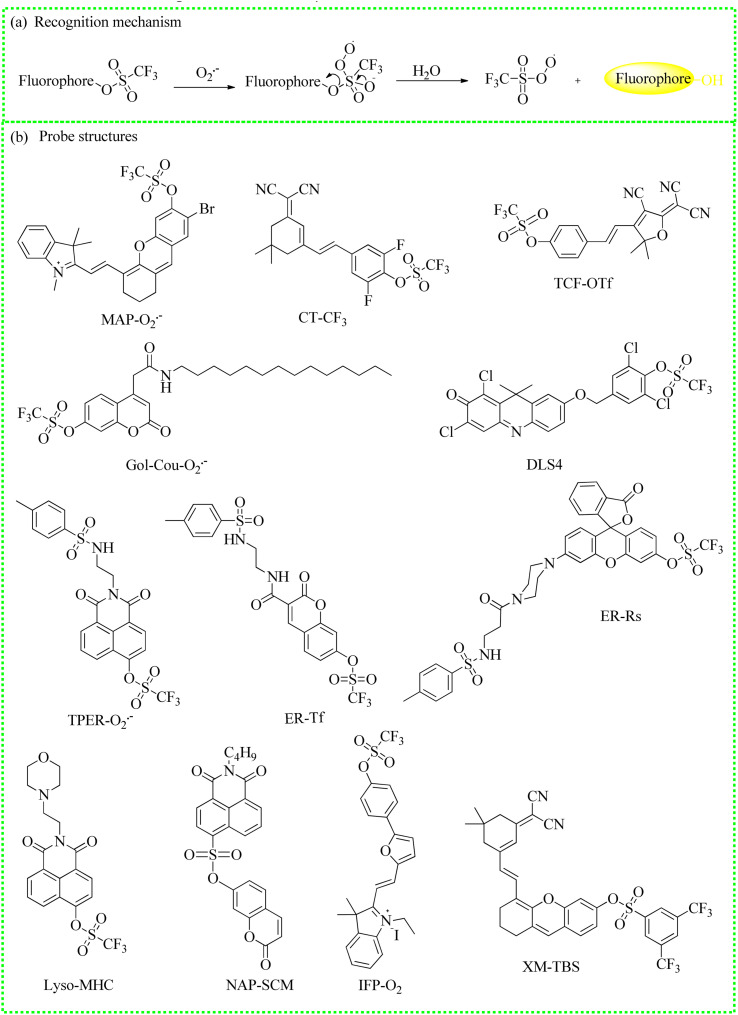
(a) The recognition mechanism between trifluoromethanesulfonate ester and O_2_˙^−^. (b) The fluorescent probe structures.

**Fig. 6:**
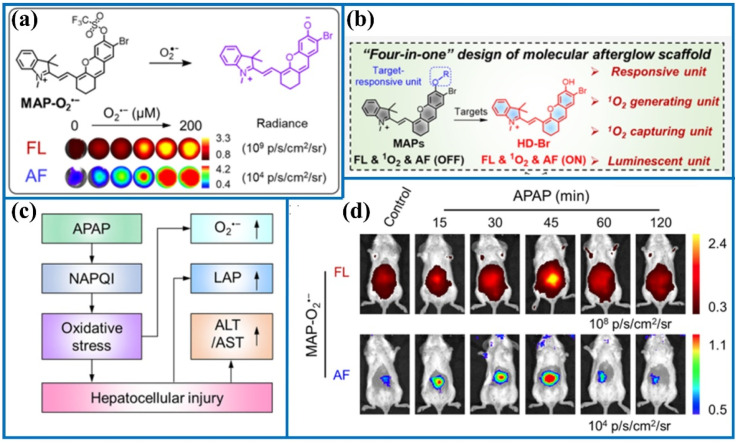
(a) The reaction principle of MAP-O_2_˙^−^ after activation by O_2_˙^−^. (b) The “four-in-one” probe design. (c) The mechanism of APAP-induced hepatotoxicity. (d) Representative fluorescence images of living mouse after the injection of MAP-O_2_˙^−^ at various time points. This figure has been reproduced from ref. 48 with permission from American Chemical Society, copyright 2023.

Zhang's group ingeniously developed an activable molecular probe (MAP-O_2_˙^−^) using a hemicyanine-based molecular scaffold for comprehensive imaging and analysis of O_2_˙^−^ changes.^48^ Moreover, employing a “four-in-one” molecular design strategy, the stimulus-response unit, ^1^O_2_ generation unit, ^1^O_2_ capture unit, and fluorophore were legitimately integrated into a single structure (Fig. 5). Subsequently, the practical applicability of the O_2_˙^−^-activated fluorescent probe was evaluated under oxidative stress. The fluorescent evidence confirmed that MAP-O_2_˙^−^ enabled super-resolution and noninvasive imaging of O_2_˙^−^ in drug-induced hepatotoxicity (Fig. 6). Furthermore, this imaging strategy identified liver toxicity earlier than serological and histological methods. Therefore, these proofs fully demonstrated the application potential of MAP-O_2_˙^−^ for the early diagnosis of hepatotoxicity.

Wang's group wisely developed a new NIR O_2_˙^−^-triggered fluorescent probe (CT-CF_3_) with a substantial Stokes shift.^49^ CT-CF_3_ rapidly reacted with O_2_˙^−^ without interference from other ROS/RNS. Moreover, CT-CF_3_ successfully monitored variations in the O_2_˙^−^ levels in different cell models using a confocal fluorescent instrument. To visualize O_2_˙^−^ in the brains, CT-CF_3_ was applied in the monitoring of neuroinflammation and schizophrenic mice under NIR excitation and emission. The fluorescence signals in the brain of schizophrenia mice were significantly higher than that of the control group since neuroinflammation and microglial cells generated the overdose of O_2_˙^−^ fluxes under oxidative stress. In summary, CT-CF_3_ not only effectively crossed the blood–brain barrier but also had capability of revealing the correlation between O_2_˙^−^ and oxidative stress. Moreover, the study introduced a screening method to evaluate the efficacy of two antipsychotic drugs. Overall, CT-CF_3_ demonstrated significant promise in bioanalytical and medical diagnostic applications.

A fluorescent probe (TCF-OTf) was effectively used to monitor the production of O_2_˙^−^ in bacteria.^50^ The reaction between TCF-OTf and O_2_˙^−^ resulted in a bright fluorescence. The reacting solutions exhibited a distinct colorimetric variation ranging from yellow to purple (aqueous phase) or blue (organic phase). In addition, with its excellent biocompatibility, TCF-OTf effectively determined the changes in O_2_˙^−^ levels in various bacteria species, such as *Pseudomonas aeruginosa*, *Staphylococcus aureus*, and *Escherichia coli*, under chloramphenicol- and heat shock-induced stress conditions.

He's group reasonably designed a new Golgi-targeting fluorescent probe (Gol-Cou-O_2_˙^−^) to rapidly detect O_2_˙^−^ in biological systems.^51^ The Gol-Cou-O_2_˙^−^ displayed numerous detection features in the buffer solution, such as a low limit of detection (3.9 × 10^−7^ M). Moreover, intracellular colocalization experiments demonstrated that the red fluorescence of Gol-Cou-O_2_˙^−^ closely overlapped with the green fluorescence of the commercial Golgi-tracker dye. Furthermore, to explore the biological feasibility of Gol-Cou-O_2_˙^−^, the exogenous and endogenous O_2_˙^−^ fluxes were consecutively visualized under various experimental conditions. Additionally, Gol-Cou-O_2_˙^−^ was employed to capture the upregulation of O_2_˙^−^ levels in myocardial cell during ischemia-reperfusion (I/R). These direct imaging findings and flow cytometry consistently demonstrated that Golgi-phosphorylated protein 3 (GOLPH3) was a potential biomarker of Golgi stress and played a crucial role during I/R. In brief, silencing GOLPH3 with siRNA resulted in decreased levels of O_2_˙^−^ and reduced apoptosis in myocardial cells during I/R. Therefore, targeting GOLPH3 may represent a novel therapeutic approach for myocardial I/R injury.

Song's group ingeniously designed a series of fluorescent sensors by attaching an NIR fluorophore to an O_2_˙^−^-sensing group (trifluoromethanesulfonate). These sensors enabled the highly sensitive and selective detection of O_2_˙^−^ levels.^52^ The stability of the probe was effectively enhanced by introducing a self-immolative linker. Subsequently, the cytotoxicity induced by doxorubicin was evaluated in H9c2 cells using the probe (DLS4). The fluorescence of phorbol-12-myristate-13-acetate (PMA)-stimulated RAW 264.7 cells increased gradually and was proportional to the dose of PMA. Ultimately, this study provided a reliable strategy for the molecular design of O_2_˙^−^-activated small-molecule fluorescent probes, which could advance the diagnosis of O_2_˙^−^-associated diseases.

Zuo's group ingeniously constructed a novel fluorescent probe (TPER-O_2_˙^−^) by integrating a two-photon fluorophore (1,8-naphthalimide), targetable moiety (*p*-methylbenzene sulfonyl chloride), and sensing unit. This probe enabled the high-fidelity detection of O_2_˙^−^ in the endoplasmic reticulum.^53^ Similarly, Dong's group synthesized two fluorescent probes (ER-Tf and ER-Rs) using coumarin and rhodol to detect changes in the O_2_˙^−^ levels in the endoplasmic reticulum, respectively.^54,55^ By substituting *p*-methylbenzene sulfonyl chloride unit with a morpholine group, Lin's group obtained a new probe (Lyso-MHC) for two-photon fluorescence mapping O_2_˙^−^ fluctuations in lysosomes, zebrafish, and pneumonia tissues.^56^ Meng's group also designed a high-quality NIR fluorescent probe (IFP-O_2_) to monitor mitochondrial O_2_˙^−^ in oral cancer cells.^57^

Using a protection–deprotection reaction strategy, Chen's group developed a novel fluorescent probe (NAP-SCM) for sensing O_2_˙^−^ fluctuations in biological systems.^58^ In the probe design, coumarin acted as the fluorescence platform and 1,8-naphthalimidesulfonyl as the masking group. NAP-SCM exhibited characteristic features for detecting O_2_˙^−^ in the buffer solution, including high specificity, rapid response (about 5 min), and a low limit of detection. With satisfactory results, such as negligible cytotoxicity, good biocompatibility, and excellent detection capabilities, NAP-SCM effectively detected fluctuations in O_2_˙^−^ levels in zebrafish embryos. Therefore, this study provided valuable insights into the roles of O_2_˙^−^ in biological processes.

A novel O_2_˙^−^-sensing NIR fluorescent probe (XM-TBS) was skillfully synthesized.^59^ Under an excitation of 690 nm, the reacting solutions exhibited significant fluorescence emission at 821 nm following the rapid reaction of XM-TBS with O_2_˙^−^. Furthermore, XM-TBS demonstrated high sensitivity toward O_2_˙^−^ with a low limit of detection of approximately 33.8 nM in fluorescence calibration experiments. Moreover, XM-TBS enabled the smooth observation of intracellular O_2_˙^−^ changes. To investigate the imaging feasibility of XM-TBS *in vivo*, XM-TBS was applied to map O_2_˙^−^ levels in mice with trazodone-induced liver injury and cyclophosphamide (CYP)-induced interstitial cystitis. The corresponding results showed that NIR emission signals increased proportionally with higher doses of trazodone, indicating a significant elevation in O_2_˙^−^ levels. More importantly, XM-TBS reliably monitored the extent of CYP-induced damage through NIR fluorescent signals, highlighting its potential for medical diagnosis and severity assessment of interstitial cystitis.

Introducing different identification mechanisms, this section reviewed the current development in the establishment of novel O_2_˙^−^-responsive fluorescent probes, such as oxidation and nucleophilic reactions. Moreover, the O_2_˙^−^ concentrations in specific locations of the cell were also accurately tracked, such as the mitochondria and endoplasmic reticulum. Among them, the dynamic and reversible detection of O_2_˙^−^ was realized with the help of DCP. Furthermore, using these fluorescent imaging tools, the alterations in O_2_˙^−^ level could be clearly detected *in situ* in certain diseases. Additionally, the O_2_˙^−^ levels were clearly observed in the deep tissue of the liver of APAP-induced early drug-induced hepatotoxicity. For instance, the activatable probes prepared by Zhang's group allowed the non-invasive imaging of hepatotoxicity before serological and histological manifestations, indicating that these probes were promising for the early diagnosis of hepatotoxicity. Particularly, the mechanism of inactivation of key proteins and the potential mechanism of depression was revealed in the combination of the probe (TCP) and proteomic analysis. Noteworthily, the recognition mechanism of these fluorescent probes and O_2_˙^−^ should be fully explained in future work. Besides, the fluorescence response of probes to other reactive specie was investigated under simulated physiological concentration. In short, these fluorescence probes may be regarded as powerful tools to detect the O_2_˙^−^ changes, assess the damage extent of the disease, study the disease mechanism and screen for drugs of the disease.

### 2.2 Monitoring of NO

NO is a highly diffused free radical, synthesized by l-arginine, NADPH, and oxygen, catalyzed with the help of different nitric oxide synthases. To date, NO has been recognized as the primary gaseous neurotransmitter and signaling molecule within the cardiovascular systems, which is crucial for regulating smooth muscle relaxation and vasodilation.^60,61^ Given its small size, relative lipophilicity, *etc.*, NO easily penetrates the cytomembrane in a concentration-dependent manner without requiring receptors or channels. However, excessive NO influxes can lead to adverse effects, including NO poisoning, and enzyme function inhibition. Moreover, uncontrolled NO secretion accelerates the production of other RNS. For example, intracellular nitroxyl (HNO) is the one-electron reduced, protonated derivative of NO, which has high chemical reactivity. HNO reacts quickly with thiols to form disulfides or sulfinamides and then causes the inactivation of enzymes. At present, nitroglycerin and sodium nitroprusside (SNP) are widely recognized as nitric oxide donors and vasodilators.^62^ Therefore, a range of effective fluorescent probes have been developed to elucidate the ambiguous roles of NO in disease-related biochemical pathways. According to the various recognition mechanisms of NO, the reported fluorescent probes were classified into the following categories (Table 2).

**Table 2:** Some detection parameters and application of NO-responsive small molecule fluorescent probes are summarized

Probe name	Excitation wavelengths (nm)	Emission wavelengths (nm)	Targeting region	Detection limits	Biological application	References
DANPY-NO	396	556	Lysosomes	77.8 nM	In endothelial cells	63
NOP	700	455/535	Cytoplasm	19.5 ± 1.00 nM	In cells and the hippocampus in brain tissue	64
Rh-NO-P	380/800	611/457	Cytoplasm	51.3 nM	In cells and the wound healing tissue of mice	65
Golgi-NO	560	589	Golgi apparatus	45 nM	In cells	66
DCM-NO	463	661	Cytoplasm	17 nM	In cells and BLM-induced pulmonary fibrosis mice models	67
Gol-NO	360	520	Golgi apparatus	1.6 nM	In cells and PD zebrafish model	68
BML	530	590	Lysosomes	10.3 nM	In cells and zebrafish	69
Lyso-TP-NO	840	539	Lysosomes	3.3 nM	In cells and mouse brain tissues	70
NDAQ	460	542	Cytoplasm	7 ± 0.4 nM	In cells	71
AC-SA	475	525/625	Cytoplasm	4.05 nM	In cells and zebrafish	72
HC-N	808	923	—	0.18 μM	In mice	73
BDP3	365	673	Cytoplasm	14 nM	In cells and mice	74
CS-Se	680	780	Cytoplasm	28 nM	In cells and saliva samples	75
Lyso-DHP	475	535	Lysosomes	47 nM	In cells	76

#### 2.2.1 Fluorescent probes based on the *O*-phenylenediamine group

**Fig. 7:**
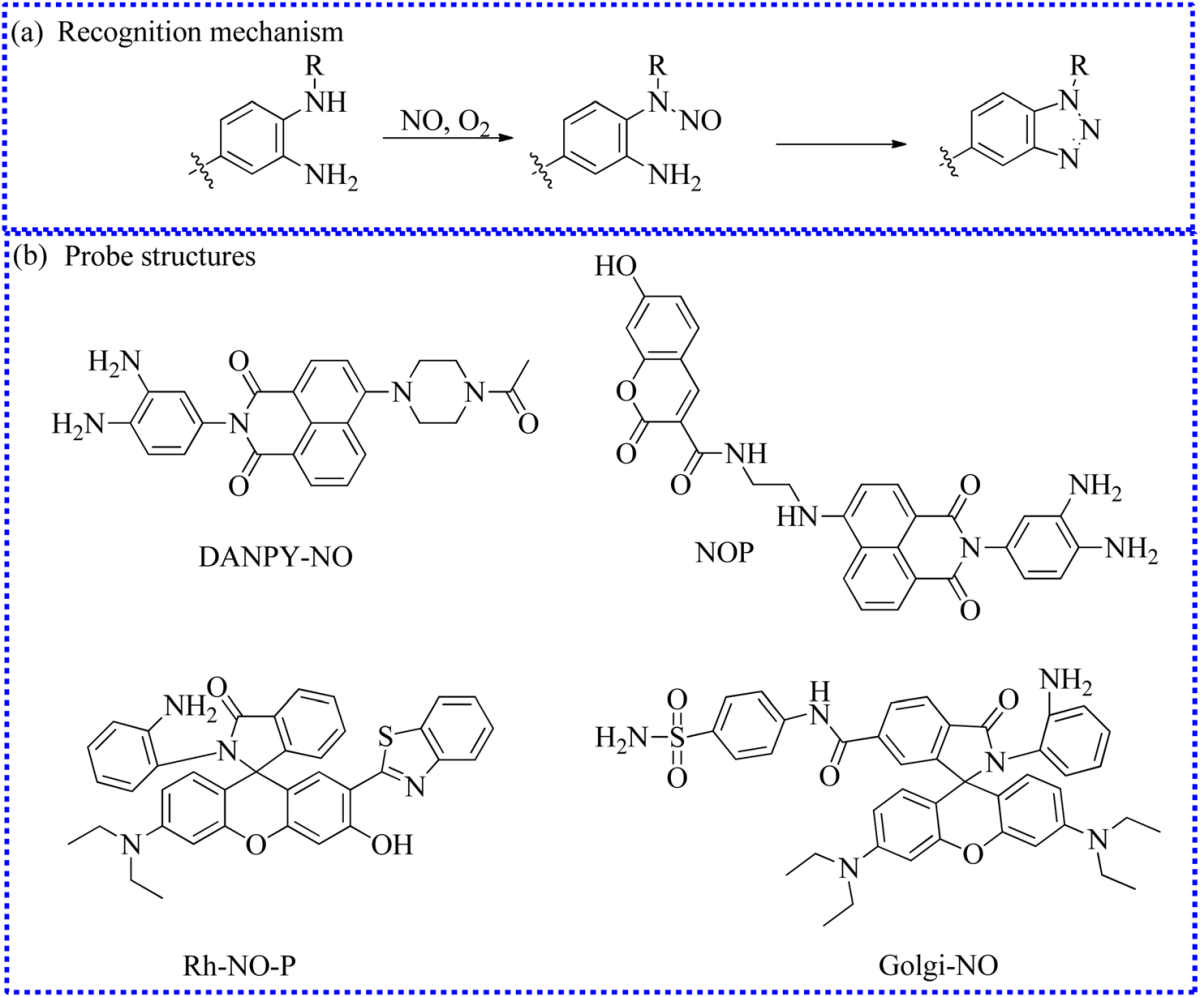
(a) The recognition mechanism between *o*-phenylenediamine and NO. (b) The structures of probes based on the *o*-phenylenediamine group.

A novel two-photon fluorescent probe (DANPY-NO) has been reported for the real-time detection of NO levels in living cells (Fig. 7).^63^ The probe itself possessed a strong absorption band at 396 nm and emission peak at 535 nm. Upon reacting with NO, DANPY-NO quickly reacted with *o*-phenylenediamine to form benzotriazole, showing an evident emission at 556 nm. Moreover, DANPY-NO identified NO with high selectivity in aqueous solution (13% DMSO). The NO levels in different types of cells were visualized successively using DANPY-NO, such as endothelial cells. Taking full advantage of the two-photon properties of 1,8-naphthalimide, the dynamic changes of intracellular NO levels were also successfully achieved under different experimental states, mainly including IFN-γ- and LPS-induced oxidative stress. The probe may be regarded as a decent fluorescent tool for the detection of NO under oxidative stress.

To quantify NO fluxes in biological systems, Tian's group developed a new two-photon ratiometric fluorescent probe (NOP) that integrated FRET and photoinduced electron transfer (PET) mechanisms.^64^ The probe design utilized coumarin and 1,8-naphthalimide as the energy donor and receptor, respectively. Significant fluorescence enhancement at 455 nm was observed in buffer solutions containing NOP and NO. However, a bright fluorescent signal at 535 nm was exhibited in the reacting solutions within 15 s. The reactions of *o*-phenylenediamine with NO generated a triazole structure, activating the FRET process and inhibiting the PET mechanism. Fluorescence emission at 455 nm gradually decreased, while there was a sharp increase in the fluorescence signal at 535 nm. Based on the two separated fluorescence peaks, NO fluxes in biological systems were quantified under two-photon excitation at 700 nm. Notably, NOP was consecutively exploited for the high signal-to-background identification of NO content in different regions of neural stem cells and mouse brain tissue with a penetration depth of 350 mm.

To further achieve quantitative visualization of intracellular NO concentrations, Song's research group developed a two-photon fluorescence probe (Rh-NO-P).^65^ By leveraging the ESIPT mechanism and rhodol ring opened-closed strategy of Rh-NO-P, the NO content was quantitatively determined using a ratiometric readout at 611 nm and 457 nm. Moreover, Rh-NO-P demonstrated several advantages, including rapid response (20 s), high sensitivity (51.3 nM), and significant fluorescent enhancement (110.9 times). Impressively, NO has been recognized as an inflammatory target in previous reports. Authors successfully tracked NO fluctuations in wound inflammation models using Rh-NO-P. Moreover, cell death and ensure wound healing were controlled by inhibitors.

**Fig. 8:**
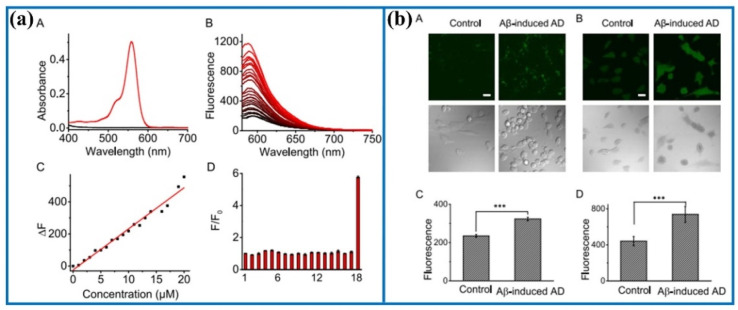
(a) Absorption and fluorescence spectra of Golgi-NO after adding NO. (b) Confocal fluorescence images of Golgi-NO in the cellular AD model. This figure has been reproduced from ref. 66 with permission from American Chemical Society, copyright 2022.

Similarly, Ma's group also developed a new fluorescent probe (Golgi-NO) to detect NO levels in Golgi apparatus.^66^ In the probe structure, the Golgi targeting group (4-sulfamoylphenylamide), classical fluorophore (6-carboxyrhodamine B), and NO recognition unit (*o*-phenylenediamine) were effectively combined to create the targeting probe (Fig. 8). Moreover, the fluorescence intensity of Golgi-NO displayed a strong linear relationship with increasing NO levels in the buffer solution. Due to the introduction of 4-sulfamoylphenylamide, the Golgi-NO exhibited superior Golgi apparatus targeting ability in the colocalization experiments. Importantly, Golgi-NO and fluorescent imaging revealed excessive NO levels in the Golgi apparatus of Aβ-induced cells. Based on the above findings, the authors speculated that increased NO levels might enhance *S*-nitrosylation and *S*-transnitrosylation, leading to synapse loss under Golgi stress.

#### 2.2.2 Fluorescent probes based on 4-(4-nitrophenyl)-thiosemicarbazide

**Fig. 9:**
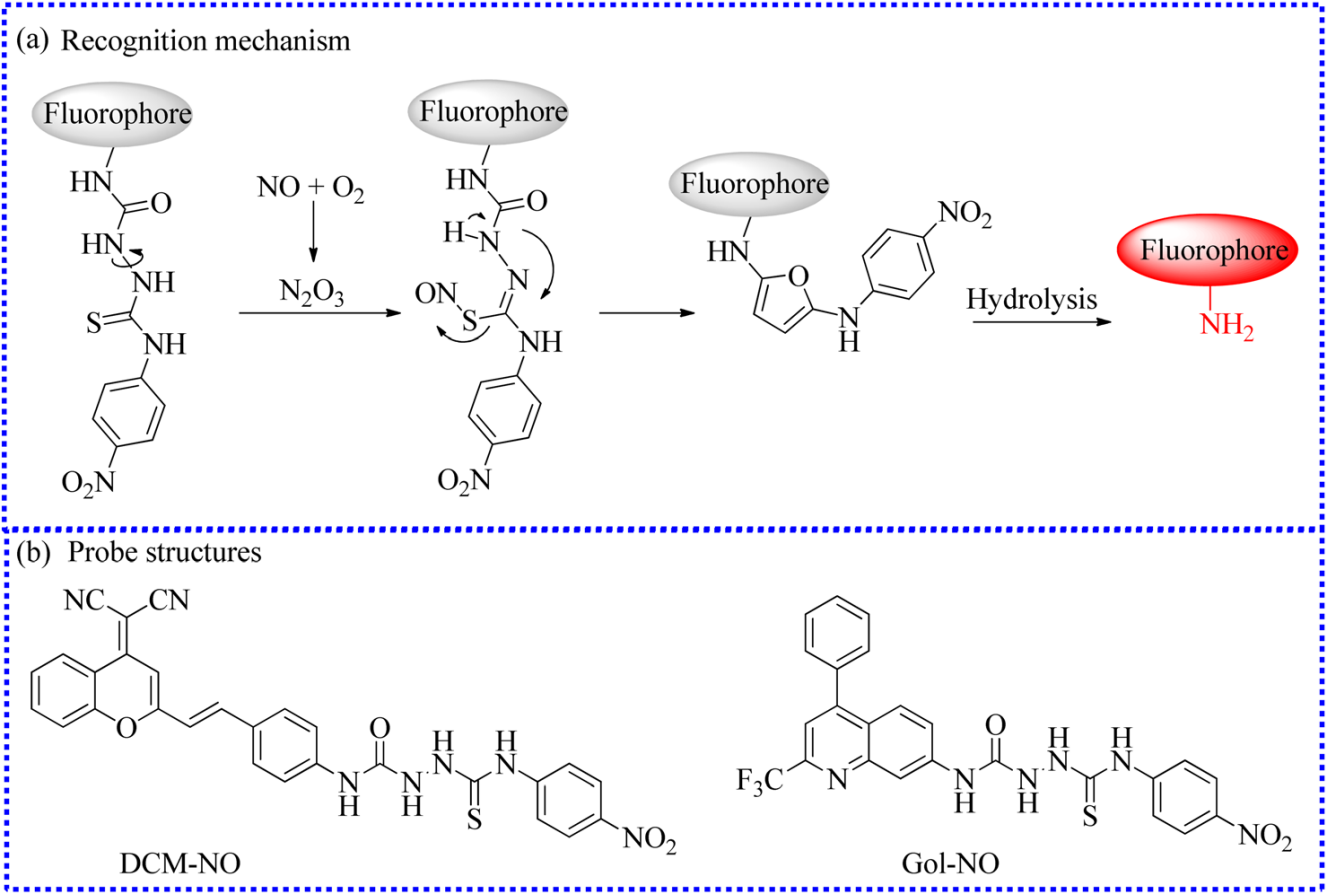
(a) The recognition mechanism between 4-(4-nitrophenyl)-thiosemicarbazide and NO. (b) The fluorescent probe structures based on 4-(4-nitrophenyl)-thiosemicarbazide.

Zhang's group elaborately created a new NIR fluorescent detector (DCM-NO) by grafting 4-(4-nitrophenyl)-thiosemicarbazide onto a DCM fluorophore.^67^ DCM-NO showed weak fluorescence in PBS because the molecular rotor of the 4-(4-nitrophenyl)-thiosemicarbazide unit freely rotated and dissipated energy (Fig. 9). After exposure to NO, the moiety was specifically sensed and cleaved, then formed a fluorophore (DCM-NH_2_) along with illuminous fluorescent signals. The whole reaction process was completed within 60 s. NO firstly reacted with this sensing group to form oxadiazole, which was rapidly hydrolyzed into the fluorophore in the identification process. The researches were pleased to directly visualize the burst of NO fluxes in pulmonary fibrosis cells and an idiopathic pulmonary fibrosis mouse model through fluorescence imaging results.

To visualize NO in Golgi, Feng's group fabricated a highly selective probe Gol-NO,^68^ which displayed many charming characteristics for NO *in vitro* experiments, such as high sensitivity (limit of detection about 1.6 nM) and fast response (<1 min). On account of the satisfactory biocompatibility and precise Golgi localization capability, Gol-NO was applied in imaging NO fluctuations in Parkinson's disease (PD) cell model. The fluorescence of PD cells was monotonously higher than that of the control group, proving that PD cells generated a certain amount of NO. Subsequently, Gol-NO was employed as a selective detection tool to image NO levels in rotenone-induced zebrafish PD models. The authors successfully observed the same experimental phenomenon.

#### 2.2.3 Fluorescent probes based on the secondary amine group

**Fig. 10:**
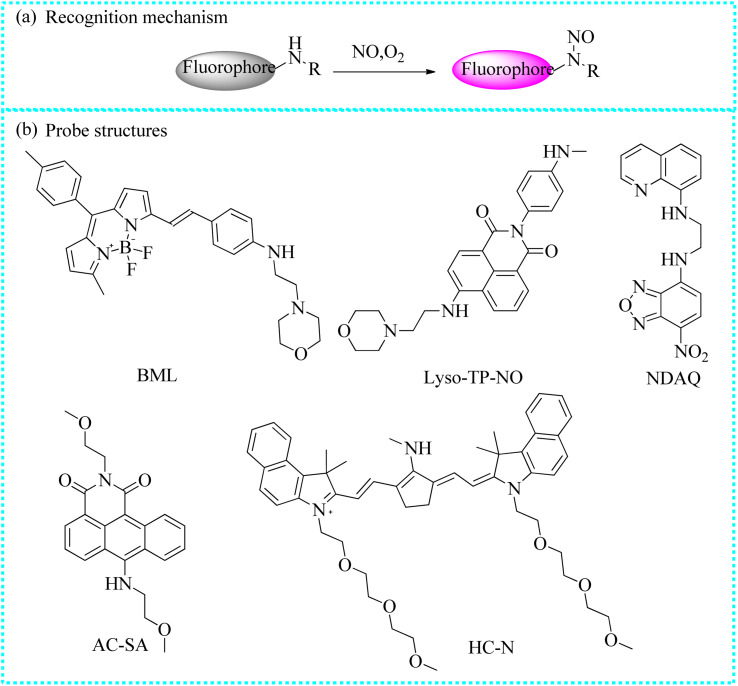
(a) The recognition mechanism between fluorescent probes and NO under O_2_. (b) The fluorescent probe structures based on the secondary amine group.

To further investigate the biological characters of NO in lysosomes, Fu's group ingeniously developed a new lysosome-targeting probe (BML) (Fig. 10).^69^ The secondary amines in the probe underwent an *N*-nitrosation reaction with NO, inhibiting the PET process and yielding a luminous production. Moreover, BML exhibited distinct characteristics for detecting NO in aqueous solution, such as rapid response (about 200 s). Moreover, BML also precisely localized to the lysosomes and monitored alterations in the NO levels in live cells stimulated with LPS. Remarkably, the intracellular fluorescence increased progressively with an excess of time, indicating a persistent rise in NO levels. Given its outstanding features, BML was employed to investigate endogenous NO influxes in zebrafish. The fluorescence intensity of zebrafish cultured with LPS or nitroprusside significantly increased. Conversely, the fluorescence intensities declined sharply after these stimulated-zebrafish were treated with L-NNA. From the above, BML had the ability of detecting the fluctuations of NO in small animals.

Liu's group also reported an “off–on” two-photon fluorescent probe (Lyso-TP-NO) for mapping lysosomal NO.^70^ Lyso-TP-NO was composed of 4-ethylamino-1,8-naphthalimide, *N*-methylaniline, and morpholine moieties. The fluorescence intensity of the reaction of Lyso-TP-NO with NO reached a stable state within 50 s. Moreover, Lyso-TP-NO exhibited a satisfactory two-photon action cross-section value (200 GM) upon the addition of NO and excitation at 840 nm. Due to the embedding of the morpholine group, Lyso-TP-NO was able to gather into the lysosome. Over time, the fluorescence intensity also increased steeply in the oxygen-glucose deprivation/reperfusion model. Taking advantage of two-photon fluorescent imaging, Lyso-TP-NO successfully detected NO levels in the mouse brain. Control mice exhibited a weak fluorescence signal. In contrast, a strong fluorescence signal was observed in the brain tissues after 1 or 2 days of cerebral ischemia, indicating there were a mass of NO influxes.

**Fig. 11:**
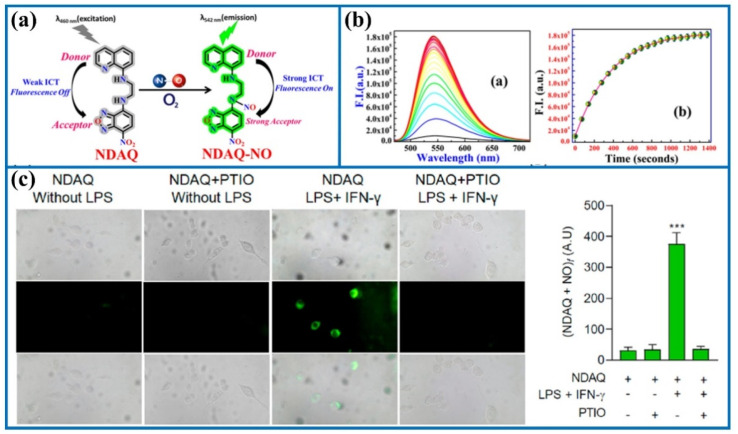
(a) The sensing mechanism of NDAQ after the addition of NO. (b) Fluorescence spectra of NDAQ after adding NO. (c) Cell confocal fluorescence images of NDAQ under treatment with stimulus in live cells. This figure has been reproduced from ref. 71 with permission from American Chemical Society, copyright 2023.

Tang's group utilized *N*-nitrosation reactions to design an organic fluorescent sensor (NDAQ).^71^ The reaction solution containing NDAQ and NO exhibited a bright fluorescent signal at 542 nm (enhanced at approximately 27 times) compared to the blank probe. Furthermore, there was a positive correlation between the fluorescence values and probe concentrations in the fluorescence measurement. Furthermore, NDAQ exhibited satisfactory anti-photobleaching effect. Additionally, confocal imaging experiments also revealed strong fluorescence in these cells upon stimulation with LPS and IFN-γ (Fig. 11), demonstrating that abundant NO levels were generated in these stimulated cells. However, upon the addition of 2-phenyl-4,4,5,5-tetramethylimidazoline-1-oxyl-3-oxide (a NO scavenger), the intracellular fluorescence was restrained. Therefore, NDAQ may serve as a reliable instrument for mapping endogenous NO variations.

To quantify NO levels, Wang's group designed a new ratiometric luminescent probe (AC-SA).^72^ The obtained probe emitted a bright red emission at 625 nm due to a strong intramolecular charge transfer (ICT) process. However, NO directly reacted with aromatic secondary amines to form electron-absorbing *N*-nitroso, resulting in the suppression of red fluorescence and enhancement of green fluorescence (at 525 nm). Moreover, there was a straightforward linear correlation between the ratiometric intensity (*I*_525_/*I*_625_) readouts and NO levels. Importantly, the NO influxes were quantitatively measured in cells and zebrafish under diverse stimulus *via* this ratiometric mode.

Wu *et al.* designed a new NO-activated probe (HC-N) for imaging acute inflammation in mice using the second near-infrared (NIR-II) fluorescence and optoacoustic imaging.^73^ In the heptamethine cyanine, two hydrophilic and biocompatible tri(ethylene glycol) chains were strategically introduced into the heterocyclic to enhance its water solubility and biocompatibility. At the same time, a secondary amine was incorporated into the heptamethine cyanine for the identification of NO. The resulting probe exhibited strong absorption at 660 nm and a negligible NIR-II fluorescence. After reacting with NO, the electron-donating methylamine was transformed into an electron-absorbing methyl-*N*-nitro group through *N*-nitrification and a new product was obtained, resulting in a significant absorption peak of 865 nm and higher fluorescence at 923 nm. Moreover, the detection ability of HC-N was evaluated in mouse models of LPS-induced acute dermatitis and monosodium iodoacetate-induced acute inflammation.

#### 2.2.4 Fluorescent probes based on other recognition groups

**Fig. 12:**
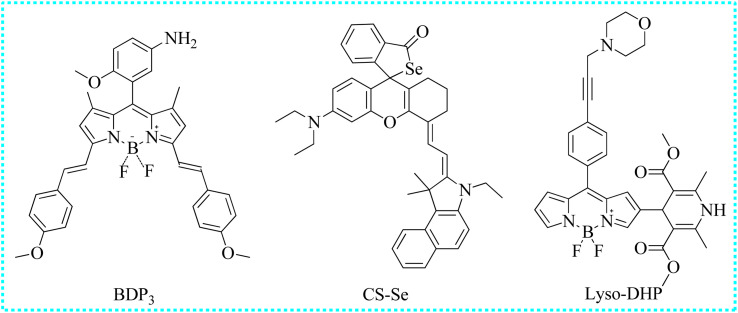
The fluorescent probe structures based on other reaction types.

Gao's group skillfully developed an NO-activated fluorescent probe (BDP3) to detect the antitumorigenic phenotype of tumor-associated macrophages (Fig. 12).^74^ Initially, BDP3 exhibited a faint fluorescence since the *para*-methoxyanilin moiety effectively inhibited fluorescence through a PET mechanism. However, the amino unit of BDP3 reacted with NO to produce a new product that exhibited bright fluorescence since the PET mechanism was eliminated. Due to its excellent benefits, such as rapid response (<1 min), resistance to interference and good biocompatibility, BDP3 was consistently used to detect the exogenous and endogenous NO fluctuations. These fluorescence signals showed a strong correlation with the tam phenotype in the tumor tissue. Therefore, the detection platform of BDP3 could evaluate the effectively targeted macrophage immunotherapy in living animals.

Utilizing this Changsha dye, Jiao's group intelligently designed an NIR fluorescent biosensor (CS-Se) for the recognition of NO concentrations.^75^ Before reacting with NO, CS-Se exhibited weak NIR fluorescence due to the closed-loop structure of Rho. Upon reaction with the Se-bond and NO, a new fluorescent product with an open-loop structure was formed. The recognition principle was further validated using high-resolution mass spectrometry. Using CS-Se, in the real sample, the NO concentrations were significantly different in the saliva of healthy people and those affected by periodontitis and oral cancer. The NO concentrations in the saliva of patients with cancer were higher than others. Moreover, the imaging capability of CS-Se to detect endogenous NO levels in oral cancer cells was further examined. Following the addition of 3-(aminopropyl)-1-hydroxy-3-isopropyl-2-oxo-1-triazene, the red fluorescence intensity of Cal-27 cells significantly increased and reached a table platform within 15 min. The experimental phenomenon showed that stimulated cells produced a substantial amount of NO. In conclusion, CS-Se may serve as a valuable tool for imaging NO variations and the early diagnosis of oral diseases.

Lu's group developed a new dihydropyridine-based fluorescent sensor (Lyso-DHP) for the highly selective monitoring of NO levels in lysosomes by modifying the BODIPY-derived Hantzsch ester.^76^ The fluorescence of the obtained probe was repressed due to the existence of the PET process. However, upon the specific recognition of NO, a bright fluorescent enhancement (140 times) was observed within 10 min. Moreover, Lyso-DHP exhibited several outstanding traits toward NO, such as ultrahigh “turn-on” fluorescent ratio and high sensitivity. The incorporation of the morpholine unit facilitated the accumulation of Lyso-DHP in the lysosomes. These induced cells emitted pronounced fluorescence in the presence of bacterial LPS and IFN-γ. Nevertheless, the intense fluorescence of these cells was obviously restricted after treatment with the inducible nitric oxide synthase inhibitor (L-NNA). Hence, Lyso-DHP was able to monitor the NO levels in subcellular locations.

These works provide a comprehensive introduction to the synthesis, distribution, biological function and changes of NO, especially in the occurrence and development of certain diseases, such as Alzheimer's disease. Among them, some ratiometric probes displayed more fascinating advantages, accurately determining the relationship between the concentration changes of NO and the damage degree. For instance, utilizing FRET and PET, Tian's group developed the two-photon fluorescent probe (NOP) for detecting and quantifying biomolecules in living cells, tissues and even the brain of AD mice. In addition, owing to the characteristics of NIR emission, the probe (DCM-NO) could rapidly diagnose the development of pulmonary fibrosis at an early condition, helping to enhance effective therapy. In addition, compared with short wavelength-emitting fluorescent sensors, the NIR fluorescence probes have the advantage of stronger tissue penetration, showing potential imaging applications of reactive species *in vivo*, especially NIR-II fluorescent probes. NIR-II fluorescent probes are urgently needed to be synthesized for obtaining accurate information of NO in deep tissues. Hence, the biological applications of probes had been extensively studied in sensing and diagnostic fields, such as HC-N. Notably, the roles of O_2_ in the reaction between some probes and NO should be elucidated. Taken together, these fluorescent sensors may be capable of monitoring NO variations and diagnosing related disease.

### 2.3 Monitoring of ONOO^−^

**Fig. 13:**
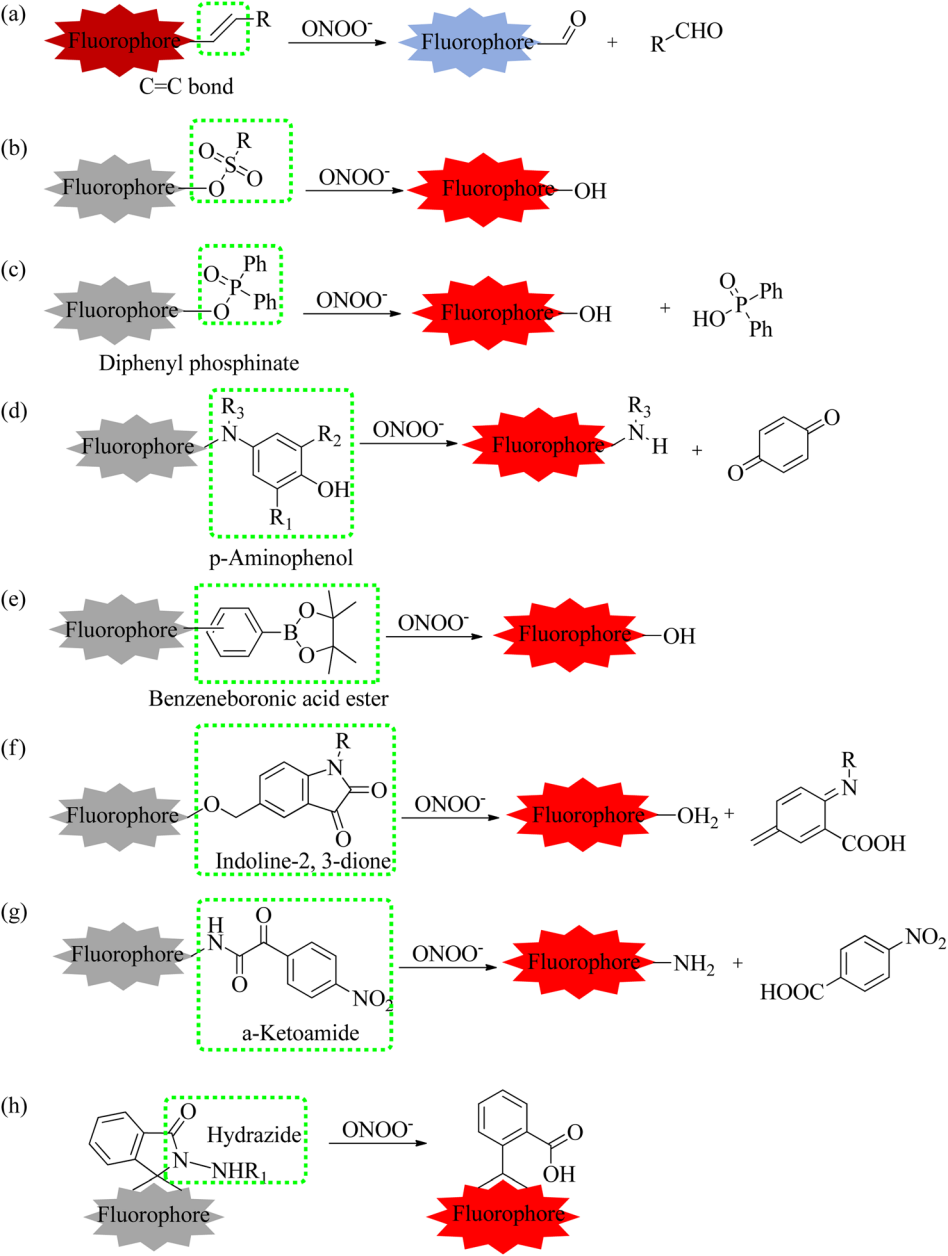
Representative types of reaction of fluorescent probes with ONOO^−^.

ONOO^−^, an endogenous RNS in living organisms, plays a significant role in multiple biological activities. Under normal conditions, ONOO^−^ acted as an essential regulator of signal transduction.^77^ However, elevated levels of ONOO^−^ oxidized or nitrated amino acid residues, such as cysteine and methionine lead to the inactivation of some proteins. For instance, the tyrosine residue was nitrated by ONOO^−^, forming 3-nitrotyrosine.^78^ Furthermore, due to stronger oxidation and nitrification, the rising levels of ONOO^−^ in phagocytes killed the invading microorganisms, which directly supported the host's immune response. In addition, the overproduction of ONOO^−^ caused irreversible damage to biological components,^79^ such as lipid peroxidation and DNA strand breakage. This damage resulted in a number of pathological conditions, mainly including apoptosis, necrosis, or ferroptosis. Recent evidence suggested that high levels of ONOO^−^ were also involved in a series of pathological processes and illnesses,^80^ such as liver damage, AD, PD, and cancer. However, the precise relationship between ONOO^−^ concentrations and related pathogenesis is unclear in the process of diseases. Therefore, advancing the accuracy measurement of ONOO^−^ can enhance our understanding of its complex roles and functions in biological systems, potentially improving the diagnosis, prevention, and treatment of ONOO^−^-associated diseases. To date, utilizing different reaction types (Fig. 13), a series of fluorescence probes have been clearly developed for ONOO^−^ monitoring, utilizing different recognition moieties (Table 3).

**Table 3:** Some detection parameters and application of ONOO^−^-responsive small-molecule fluorescent probes are summarized

Probe name	Excitation wavelength (nm)	Emission wavelength (nm)	Targeting location	Detection limit	Biological application	References
MG-ONOO	410	477/650	Cell membrane and Golgi apparatus	13 nM	In cells and in mice	81
DCO-POT	500	670	Mitochondria	10 nM	In cells, zebrafish and in the mice brain slices	82
COUS	400	484/723	Mitochondria	41.88 nM	In cells	83
SX-1	360	456	Lipid droplets	326 nM	In cells	84
TR-ONOO^−^	390	490	Cytoplasm	0.52 μM	In cells	85
NFP-ONOO	500	654	Cytoplasm	54.7 nM	In cells, zebrafish and AKI of mice	86
2F-RBH	560	715	Cytoplasm	—	In cells and a mouse model of DSS-induced colitis	87
BDPP	530	613	Cytoplasm	2.64 μM	In cells and zebrafish	88
JQ-3	523	557	Cytoplasm	32 nM	In cells and zebrafish embryo	89
Rd-DPA3	560	698	Mitochondria	3.4 nM	In cells and AD mice models	90
ER-ONOO^−^	450	557	Endoplasmic reticulum	5.2 nM	In cells and mouse organs	91
NNP	432	560	Cytoplasm	0.13 μM	In cells and acute liver injury mice model	92
DDAO-PN	600	657	Cytoplasm	50 nM/197 nM	In cells and inflammation of the mice	93
NAF-BN	600	695	Cytoplasm	436 nM	In cells and *Drosophila* brains	94
Cy-OH-ONOO	—	705	Cytoplasm	56 nM	In cells, zebrafish and mice breasts	95
L	421	500	Mitochondria	70.8 nM	In cells	96
MBDP-Py^+^	465	613	Lipid droplets	52 nM	In cells and NAFLD combined with the DILI mice model	97
Lyso-ONOO	450	555	Lysosome	0.13 μM	In cells and 4T1-xenograft tumor mice	98
Rhod-DCM-B	480	570/670	Cytoplasm	52 nM	In cells and LPS-induced living mice	99
BTNB	442	605/530	Mitochondria	0.25 μM	In cells, zebrafish and the locust Malpighian tubes	100
NIRII-HD5-ONOO^−^	—	895/936	—	—	APAP-induced liver injury mice	101
RFAc	525	590	Cytoplasm	220 nM	In cells and LPS-induced inflammation of the mouse models	102
CMONOO2	400	510	Cytoplasm	21.4 nM	In cells and in inflammation mice and tissues	103
BY-1	491	668	Lysosome	—	In cells and the collagenase-induced osteoarthritis mice models	104
HJ-ONOO-P3	582	719	Mitochondria	25.4 nM	In cells and middle cerebral artery occlusion model mice	105
Rd-PN2	500	557	Cytoplasm	—	In cells and LPS-induced kidney injury of zebrafish, mouse brain microvessels	106
SPN	680	732	Cytoplasm	19.7 nM	In cell and zebrafish and mice	107
NIR-ONOO	620	650	Cells	15 nM	In cells and mouse liver tissue slices and living mice	108
BP-ONOO	570	613	Cytoplasm	18 nM	In cells	109
RB-PN	530	575	Cytoplasm	7 nM	In cells and tumor 3D micro-spheroid	110
RH-PN	360	581/454	Cytoplasm	93 nM	In cell and zebrafish	111
Mito-NA	390	558/454	Mitochondria	0.12 μM	In cells	112
NATP	445	565	Cytoplasm	—	In cells and AD mice	113
ON-RB	620	672	Lipid droplets	6.3 nM	In cells	114

#### 2.3.1 Fluorescent probes based on the C=C bond

**Fig. 14:**
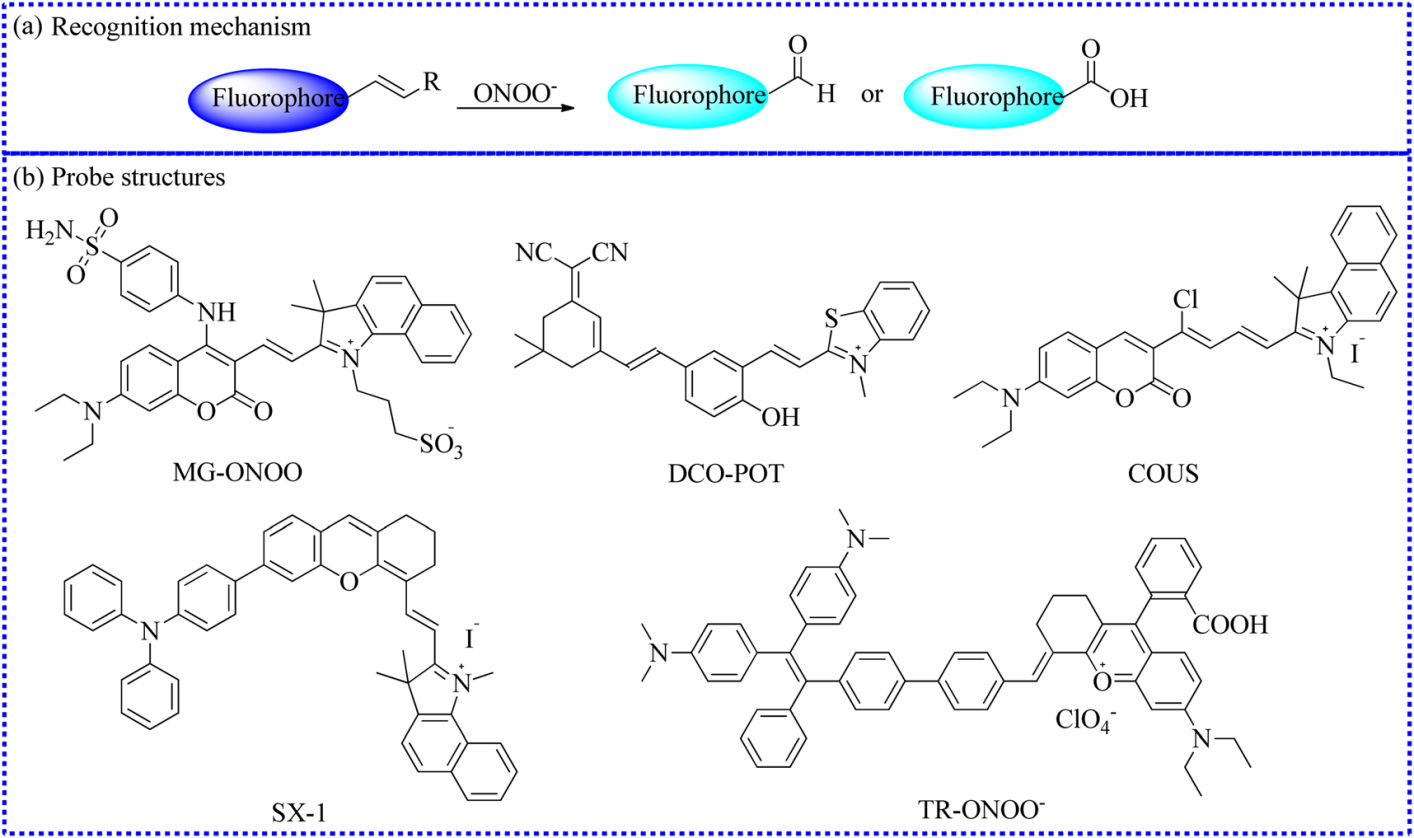
(a) The reaction mechanism between fluorescent probes based on the C=C bond and ONOO^−^. (b) The fluorescent probe structures based on the C=C bond.

To achieve the precise detection of reactive molecules, the ratiometric approach is highly effective.^115^ For example, ONOO^−^ was capable of destroying the C=C bond, transforming a molecular probe with a large conjugated structure into a small conjugated structure.^116^ To investigate the relationship between oxidative stress the Golgi apparatus and drug-induced liver injury (DILI), the Feng's group utilized an organic fluorescence probe (MG-ONOO) to image ONOO^−^ levels in the cell membranes and Golgi apparatus under different experimental conditions (Fig. 14).^81^ MG-ONOO was formed by connecting coumarin and hemi-cyanine through a C–C bond. The blank probe exhibited a strong NIR fluorescent peak at 650 nm due to the activation of the ICT effect from the large π–π conjugation. Upon incubation with ONOO^−^, the C=C bond in the molecular skeleton was specifically disrupted, along with an increasing fluorescent peak at 477 nm. The ratiometric fluorescence intensity (*I*_477_/*I*_650_ nm) was enhanced roughly 125-fold with the incremental addition of ONOO^−^. The fluorescent region of HeLa cells shifted from the cell membrane to the Golgi apparatus over time. Subsequently, under monensin-induced oxidative stress, the red channel signals significantly decreased while the blue channel signals substantially increased, indicating that the stimulated cells produced abundant ONOO^−^. To further demonstrate that these intracellular fluorescence changes were due to excessive ONOO^−^, uric acid was selected to remove intracellular ONOO^−^ effectively. The strong red fluorescent signals were significantly inhibited in monensin-induced cells, demonstrating that MG-ONOO could sensitively detect intracellular ONOO^−^. Gratifyingly, MG-ONOO was also applied to measure the dynamic variations of ONOO^−^ in the liver of DILI mice, proving that elevated levels of ONOO^−^ were closely related to the initiation and progression of DILI. Notably, this MG-ONOO-based strategy may be utilized to evaluate potential drugs for remediating DILI and exploring innovative treatment strategies.

Mitochondria are the primary sites of ONOO^−^ generation in life processes. Therefore, the accurate measurement of mitochondrial ONOO^−^ fluxes is essential for comprehensively understanding its biological functions. Therefore, Wu's group established an NIR fluorescent biosensor (DCO-POT).^82^ The 4-methylbenzothiazole cation served as a mitochondrial targeting unit. In the presence of ONOO^−^, the C=C bond in DCO-POT was specifically interrupted, leading to the rapid release of strong NIR fluorescence from the fluorophore. Moreover, DCO-POT reacted rapidly with ONOO^−^, and the fluorescence was slightly stable. The colocalization experiments confirmed that DCO-POT accurately localized within the mitochondria. Satisfactorily, the dynamic variations of ONOO^−^ were observed when the cells were treated with different stimuli. To further explore the ambiguous relationship between the pathogenesis of AD and ROS-mediated oxidative stress, DCO-POT was used as a high-contrast imaging indicator for visualizing ONOO^−^ in the brain of AD mouse models.

Based on a coumarin-cyanine hybrid, Hu's group rationally designed a novel NIR fluorescent probe (COUS) for the ratiometric detection of ONOO^−^ fluxes.^83^

To investigate the ONOO^−^ levels in lipid droplets under oxidative stress, Ye's group deftly developed a novel fluorescent probe (SX-1) employing a fluorophore (triphenylamine-benzoindocyanine) for the specific monitoring of ONOO^−^ in cyclophosphamide-induced HeLa cells.^84^ The C=C bond in the probe was specifically cleaved by ONOO^−^, releasing a benzoindolecyanine segment, which emitted blue fluorescence. Upon exposure to ONOO^−^, the fluorescence of SX-1 gradually enhanced and reached a stable platform within 3 min. Furthermore, the probe selectively identified ONOO^−^ without interference from other species. Moreover, the emission signals of SX-1 with varying ONOO^−^ fluxes were linearly fitted. SX-1 was accumulated in the lipid droplets and detected ONOO^−^ fluxes in live cells. Encouragingly, the authors provided the direct evidence of upregulation of ONOO^−^ content in HeLa cells induced by cyclophosphamide.

Similarly, by connecting a tetraphenylene derivative with rhodamine analogs, this group also developed a new fluorescent imaging tool (TR-ONOO^−^) with a large conjugated structure for detecting ONOO^−^.^85^ Intracellular fluorescent imaging results demonstrated that TR-ONOO^−^ could effectively track ONOO^−^ influxes in biological systems.

#### 2.3.2 Fluorescent probes based on the trifluoromethanesulfonate group

**Fig. 15:**
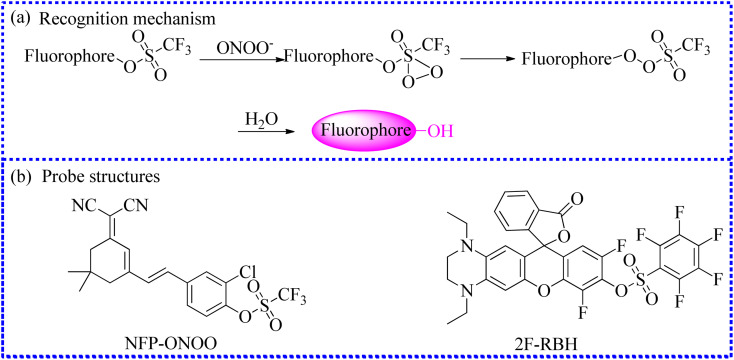
(a) The sensing mechanism between trifluoromethanesulfonate ester and ONOO^−^. (b) The fluorescence probe structures.

To comprehend the potential connections between ONOO^−^ levels and kidney injury induced by ferroptosis, Jiang's group constructed a novel NIR fluorescent sensor (NFP-ONOO) employing the ICT mechanism.^86^ The free probe was initially nonfluorescent because the OH unit in the fluorophore was sulfonated by trifluoromethanesulfonic anhydride (Fig. 15). Upon nucleophilic reaction with ONOO^−^, the recognition moiety was promptly identified and separated, allowing fluorescence improvement of the free fluorophore after 25 min. Moreover, the addition of Cl atom enhanced the fluorescence amplification of the fluorophore. NFP-ONOO exhibited good imaging capability of ONOO^−^ fluxes in acute kidney injury (AKI) cells. Fluorescent signals were significantly increased in erastin-induced cells than that in the control cells. However, the intracellular fluorescent intensity was visibly reduced after treatment with resveratrol. Moreover, the authors also observed this phenomenon in erastin-mediated AKI mice. In summary, these experiments demonstrated a good relationship between the ONOO^−^ levels and ferroptosis-mediated AKI, suggesting that this NFP-ONOO acted as a sensitive sensor to clarify the roles of ONOO^−^ in ferroptosis-mediated AKI.

The accurate diagnosis of inflammatory bowel disease (IBD) is crucial for effective treatment. Therefore, Xing's group created a series of fluorescent probes by grafting the F or Cl atom in the fluorophore.^87^ Among them, 2F-RBH exhibited several favorable properties for detecting ONOO^−^*in vitro*, such as NIR emission, excellent photostability, large Stokes shift, and pH insensitivity. Moreover, the upregulation of ONOO^−^ in IBD cells was precisely identified using 2F-RBH and confocal fluorescence instrument. Therefore, 2F-RBH can serve as an auxiliary imaging tool for the reliable diagnosis of IBD.

#### 2.3.3 Fluorescent probes based on diphenyl phosphinate group

**Fig. 16:**
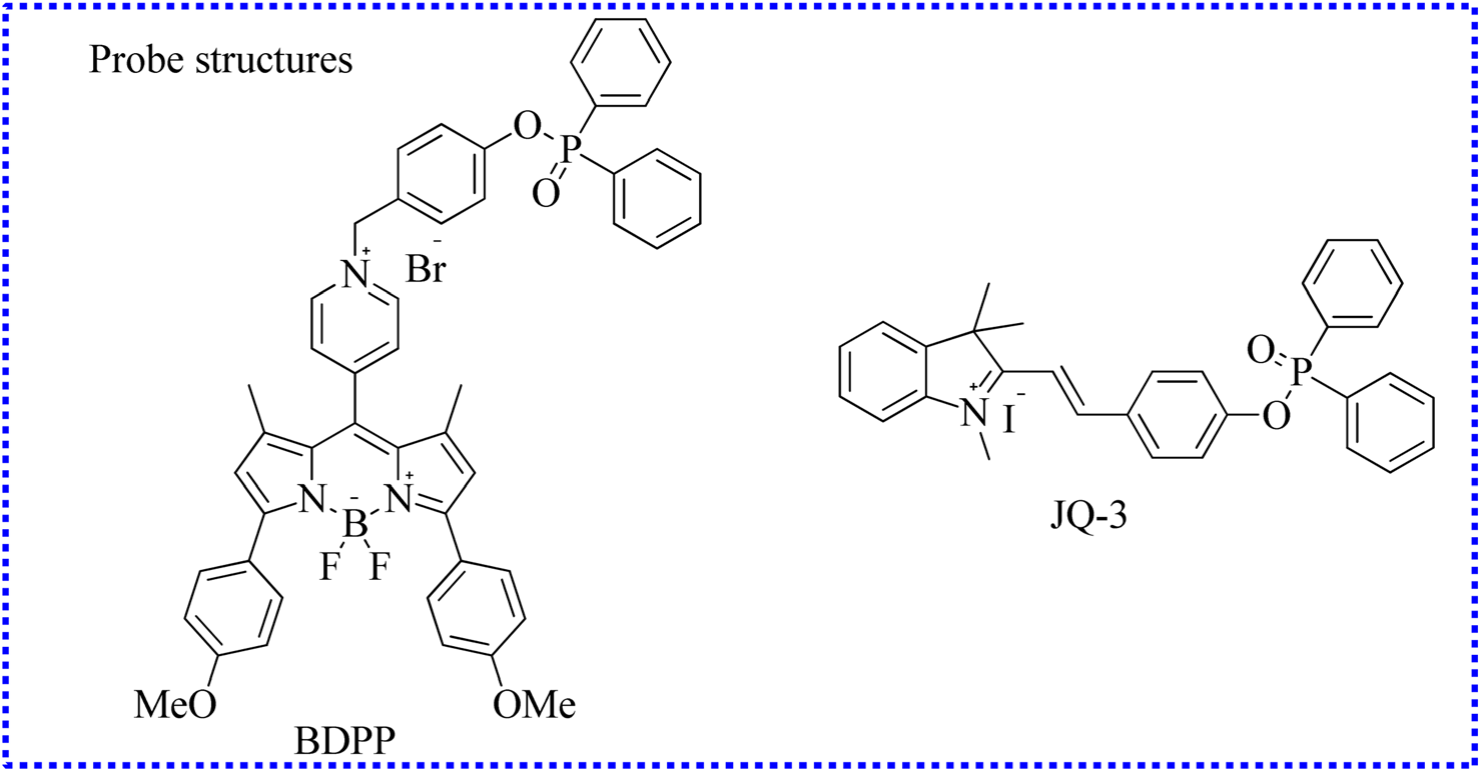
The fluorescent probe structures based on the diphenylphosphinyl group.

Zhang's group designed a new NIR fluorescent probe (BDPP) for precisely detecting ONOO^−^ levels.^88^ BDPP was made up of a BODIPY scaffold and a diphenyl phosphinate group (Fig. 16). The probe itself displayed negligible fluorescence because the OH unit in the fluorophore was caged by the trigger group. Upon stimulation with ONOO^−^, the sensing group in the BDPP was attacked, releasing the free fluorophore, which enhanced the absorption peak and fluorescence emission. In the presence of ONOO^−^, the fluorescence of this BDPP reached a stable state within 10 minutes. Therefore, BDPP effectively detected endogenous ONOO^−^ fluctuations in acetaminophen (APAP)-induced hepatotoxicity cells. Nevertheless, a weak emission signal was observed after incubation with *N*-acetylcysteine (NAC), indicating that NAC could mitigate APAP-induced hepatotoxicity to some extent. This study provided an imaging tool for the high-throughput screening of drugs in liver injury.

To further examine the ONOO^−^ levels in drug-induced hepatotoxicity, using an ICT mechanism, Kang's group selected merocyanine dye as the fluorophore and then successfully established a new small-molecule fluorescence probe (JQ-3).^89^ The imaging detection of intracellular ONOO^−^ was successfully achieved utilizing JQ-3. These imaging results further confirmed that high fluxes of ONOO^−^ were present in the DILI cell models.

#### 2.3.4 Fluorescent probes based on the *p*-aminophenol group

**Fig. 17:**
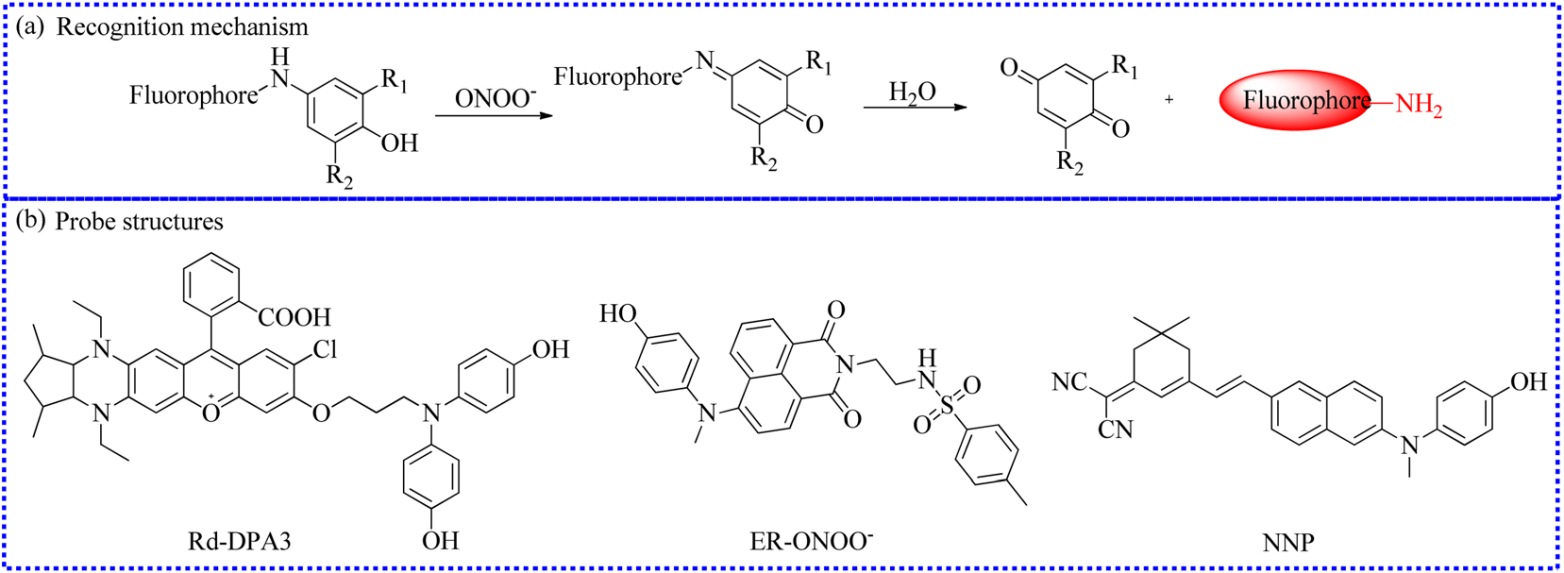
(a) Sensing mechanism between *p*-aminophenol and ONOO^−^. (b) The fluorescent probe structures based on *p*-aminophenol derivatives.

Given this knowledge, *p*-aminophenol, is easily oxidized by peroxidases.^117–119^ Currently, it has been successfully utilized for creating a variety of ONOO^−^-activated fluorescent probes.^120^ Leveraging the electron-rich nature of *p*-aminophenol, the fluorescence of these probes was effectively quenched *via* the PET process. Upon reacting with ONOO^−^, the *p*-aminophenol group in the probe structure was rapidly oxidized, liberating *p*-benzoquinone and obtaining a free fluorophore (Fig. 17).

**Fig. 18:**
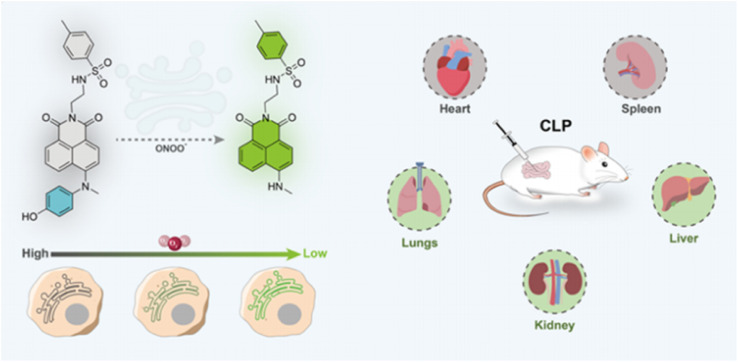
(a) Chemical structure of ER-ONOO^−^, and the fluctuations of ONOO^−^ in the endoplasmic reticulum and CLP under hypoxic conditions were detected utilizing ER-ONOO^−^. This figure has been reproduced from ref. 91 with permission from American Chemical Society, copyright 2023.

Through molecular structural regulation, 4,4′-azanediyldiphenol and Rhodol were combined to create a new fluorescent probe (Rd-DPA3).^90^ This probe specifically reacted with ONOO^−^, emitting a significant NIR fluorescence. Additionally, Rd-DPA3 possessed mitochondrial targeting capabilities, allowing it to monitor the variations of ONOO^−^ in the brain cells under oxidative stress. The small molecular structure and adjustable lipophilicity of Rhodol further endowed Rd-DPA3 with excellent blood–brain barrier (BBB)-crossing abilities. Furthermore, these specialties enabled Rd-DPA3 to monitor ONOO^−^ levels in the brain (Fig. 18). *In vivo* fluorescence imaging results showed an increase in the ONOO^−^ levels in the brains of mice with AD. Consequently, this study revealed that Rd-DPA3 was essential for the precise diagnosis and effective assessment of AD.

To examine the latent relationships between ONOO^−^ levels in the endoplasmic reticulum and hypoxia-induced endothelial injury in sepsis, Lv's group fabricated a two-photon fluorescent probe (ER-ONOO^−^).^91^ In this probe, the methyl(4-hydroxyphenyl)amino moiety served as the recognizing domain for ONOO^−^ and quenching group of the probe. The absorption wavelength of ER-ONOO^−^ remained unchanged before and after reacting with ONOO^−^. However, significant fluorescent enhancement was observed in the reacting solutions within 1 s. Due to the presence of a *p*-toluenesulfonamide group, ER-ONOO^−^ accurately targeted the endoplasmic reticulum (a colocalization coefficient of 0.934). Under hypoxic environments, intracellular fluorescence increased stepwise with the increase in CoCl_2_ content, indicating that these stimulated cells steadily produced a large amount of ONOO^−^. ER-ONOO^−^ successfully detected ONOO^−^ levels in the organs at various stages in a cecum ligation and puncture mouse model. This work based on ER-ONOO^−^ offered a valuable and universal tool for visualizing the ONOO^−^ content in the pathogenesis and treatment of sepsis.

For the early and reliable detection of acute liver injury, a novel fluorescent probe (NNP) was designed for the highly selective and rapid measurement of ONOO^−^.^92^ NNP exhibited outstanding advantages for ONOO^−^ in buffer solution, including marked color changes (red to yellow) and favorable stability. Utilizing NNP, dynamic variations of ONOO^−^ were successfully detected in living LX-2 cells under stimulation of 3-morpholinosydnonimine (SIN-1), LPS, and PMA.

#### 2.3.5 Fluorescent probes based on the benzyl borate moiety

**Fig. 19:**
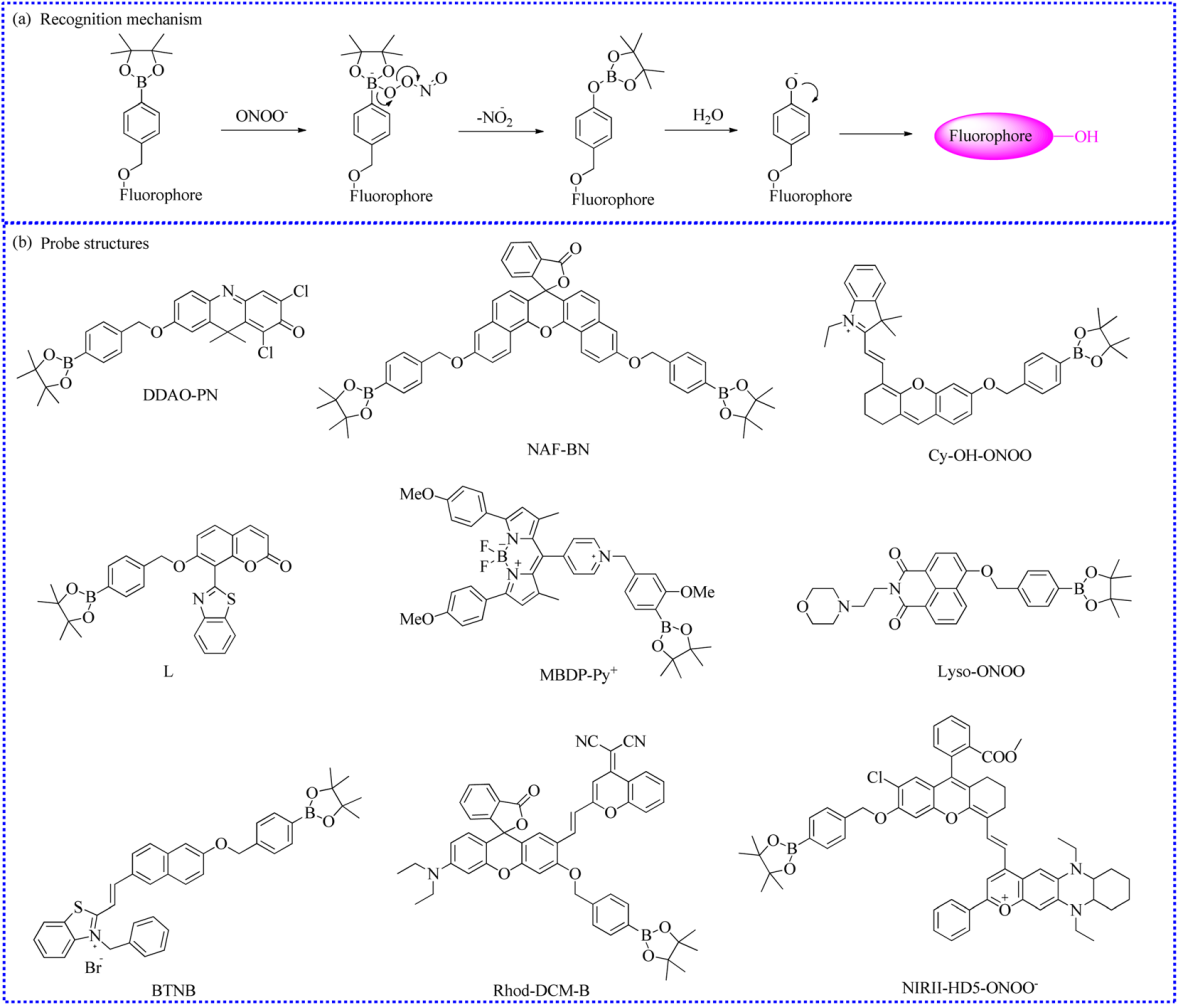
(a) The sensing reaction between benzyl borate moiety and ONOO^−^. (b) The probe structures based on benzyl borate moiety.

To clarify potential correlations between ONOO^−^ influxes and inflammatory processes, Liu's group constructed a high-performance NIR fluorescent probe (DDAO-PN) (Fig. 19).^93^ DDAO-PN specifically responded to ONOO^−^*in vitro*, producing a significant NIR fluorescence increase (approximately 84 times) at 657 nm within 30 s. Moreover, intracellular fluorescence signals (F/F_0_) showed a 68-fold enhancement, indicating that exogenous ONOO^−^ levels were smoothly elevated. Subsequently, *in vivo* fluorescent imaging experiments demonstrated that DDAO-PN could detect ONOO^−^ concentrations in LPS-induced rear leg inflammation, showing a 4.0-fold increase in fluorescence signals. The clear fluorescence enhancement and rapid response ability gave DDAO-PN a good signal-to-noise (S/N) ratio for the real-time monitoring of ONOO^−^ fluctuations. Taken together, DDAO-PN may be served as a valuable NIR fluorescent tool for imaging ONOO^−^ in inflammation. Compared with the control group, the inflammatory mice showed intense fluorescence, indicating that the inflammatory sites produced significant amounts of ONOO^−^.

A new NIR fluorescent probe (NAF-BN) was synthesized by modifying the naphthofluorescein core.^94^ ONOO^−^ was equipped to transform the fluorophore with a lactone structure into an open-loop structure. NAF-BN identified ONOO^−^ with high selectivity upon exposure to other reactive species in selective experiments. Moreover, the emission signals of NAF-BN were rapidly enhanced and positively correlated with the gradual increase in ONOO^−^ fluxes. The results of intracellular fluorescence imaging further demonstrated that cells emitted stronger red fluorescence when stimulated with SIN-1 or LPS and PMA. Upon treatment with a ROS scavenger (minocycline), the red fluorescence of SIN-1-stimulated cells was significantly restrained, implying a reduction in ONOO^−^ concentrations. Similarly, the imaging capabilities of NAF-BN were also tested *in vivo*. *Drosophila* brains treated with SIN-1 and NAF-BN showed stronger red fluorescence than the control *Drosophila* brains due to the excessive expression of endogenous ONOO^−^. Therefore, these excellent imaging performances made this probe a promising biosensor for monitoring ONOO^−^ in biological and medical fields.

An ultrafast NIR fluorescent probe (Cy-OH-ONOO) was rationally constructed to measure ONOO^−^ levels in a mouse model of breast cancer.^95^ The fluorescence of Cy-OH-ONOO steadily increased with rising ONOO^−^ concentrations, reaching a steady state. Moreover, Cy-OH-ONOO exhibited significant imaging traits. Using this Cy-OH-ONOO, researchers discovered that the fluorescence signals in mouse tumor sites were significantly stronger than those in normal tissues, indicating that high levels of ONOO^−^ were produced in tumor locations.

Adopting ICT and FRET mechanisms, a coumarin-based fluorescent probe (L) was synthesized for the rapid detection of exogenous and endogenous ONOO^−^ influxes.^96^

**Fig. 20:**
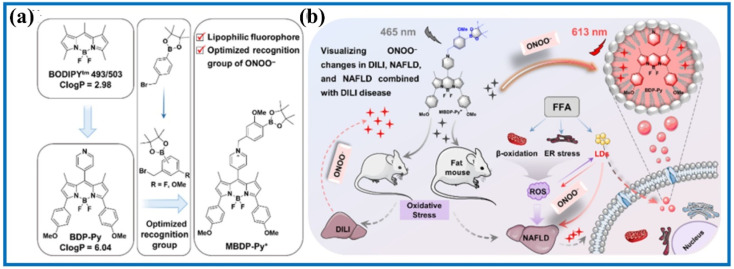
(a) Chemical structure and (b) fluorescence imaging of MBDP-Py^+^ in NAFLD and with DILI mice. This figure has been reproduced from ref. 97 with permission from American Chemical Society, copyright 2023.

Previous studies reported that liver lipid droplets and ONOO^−^ levels are closely related to nonalcoholic fatty liver disease (NAFLD). Decorating the BODIPY dye, Zhao's group constructed an ONOO^−^-activated fluorescent probe (MBDP-Py^+^) to investigate the synergistic interactions between NAFLD and DILI.^97^ MBDP-Py^+^ exhibited numerous satisfactory detection features in buffer solutions, such as fast response and high contrast. Moreover, the red channel of MBDP-Py^+^ and green channel of the commercial dye predominantly overlapped, showing a good Pearson's colocalization coefficient. Using MBDP-Py^+^ and fluorescent confocal microscopy, the ONOO^−^ levels were visualized in DILI cells, DILI mouse models, and HFD-induced NAFLD mouse models. Compared with existing evaluation methods, such as serum enzyme assays and hematoxylin and eosin staining, the MBDP-Py^+^-based dual-parameter approach enabled the early diagnosis of NAFLD and DILI by detecting the overproduction of ONOO^−^ concentrations (Fig. 20). The study also found that liver damage was mainly caused from the drug at the early stages of NAFLD combined with DILI mice models. However, the extent of NAFLD deepened over time and the synergistic effect of NAFLD and DILI increased, resulting in severe liver damage. Overall, this work contributed to a profound understanding of the pathogenesis of NAFLD combined with DILI and provided a high-throughput screening approach for the precise diagnosis of NAFLD and DILI.

To visualize lysosomal ONOO^−^, a multifunctional small-molecule fluorescent probe (Lyso-ONOO) was successfully designed.^98^ Compared with other biological species, Lyso-ONOO only detected ONOO^−^ with high sensitivity. Due to the presence of a morpholine group, Lyso-ONOO was specifically collected in lysosomes over other organelles. Importantly, Lyso-ONOO enabled the measurement of ONOO^−^ levels in three types of acute liver injury models and mice with drug-treated liver injury (silybin and bicyclol). This effective strategy based Lyso-ONOO provided an exceptional method for assessing the severity of acute liver injury and facilitating treatment discovery.

To realize the quantitative detection of ONOO^−^ during liver injury, Li's group developed a novel ratiometric sensing probe (Rhod-DCM-B) by adopting a structural modification strategy.^99^ Rhod-DCM-B consisted of a new extended π-conjugated fluorophore (dicyanomethylene-benzopyran, DCM) and a classical fluorophore (rhodamine). The free Rhod-DCM-B exhibited an obvious NIR fluorescence peak, which was ascribed to the typical emission of DCM. In the presence of ONOO^−^, the recognition group was promptly oxidized, forming an open-ring rhodamine structure and recovering strong fluorescence. Therefore, the precise testing of ONOO^−^ was achieved using a ratiometric fluorescence method at different wavelengths (570 nm and 670 nm). Notably, encouraged by these advantages, such as excellent sensitivity, high selectivity, and good biocompatibility, Rhod-DCM-B enabled the successful imaging analysis of ONOO^−^ levels in the liver of mice with DILI.

For the quantitative detection of ONOO^−^ levels in mitochondria, a mitochondrial-targeting fluorescent sensor (BTNB) was created.^100^ The molecular skeleton of the probe comprised of a benzothiazolium-naphthol hybrid and benzyl borate moiety. Meantime, the cationic benzothiazolium unit not only was acted as the mitochondrial location group but also improved the water solubility of BTNB. The maximum absorption band redshifted from 442 nm to 568 nm after BTNB reacted with ONOO^−^ in the reacting solutions. Simultaneously, the solution color also changed from yellow to pink. Additionally, pretreatment with ONOO^−^ caused a redshift of the maximum fluorescence emission from 570 nm to 605 nm. Interestingly, BTNB specifically monitored ONOO^−^ without interference from other redox species in selectivity experiments. Moreover, due to its low cytotoxicity, BTNB enabled the quantitative determination of ONOO^−^ in RAW 264.7 cells under different stimuli. Colocalization experiments confirmed that BTNB selectively entered into the mitochondria with a high overlap coefficient (0.92). Fluorescence imaging results indicated that zebrafish stimulated by SIN-1 fluoresced more brightly compared to the control zebrafish or those stimulated by SIN-1 and uric acid. Overall, this work offered a new ratiometric imaging strategy to further uncover possible molecular mechanisms of ONOO^−^ in living organisms.

To further optimize the structure of hemicyanine, decahydroquinoxaline pyranium salts replaced the indole salt group in a larger conjugated system. The stability of oxanthrene was enhanced by introducing large steric groups at position 9. Moreover, the introduction of chlorine atom at adjacent sites reduces the pKa of the hydroxyl group. The modified fluorophore exhibited long emission wavelengths (extending to the NIR-II region) and good stability. Building on this foundation, Yuan's group further constructed an ONOO^−^-activated NIR-II fluorescence probe (NIRII-HD5-ONOO^−^) using a borate structure.^101^ This probe enabled high-reliability imaging and analysis of ONOO^−^ concentrations, which was applied in sentinel lymph node metastasis and liver injury.

#### 2.3.6 Fluorescent probes based on the α-keto amide group

**Fig. 21:**
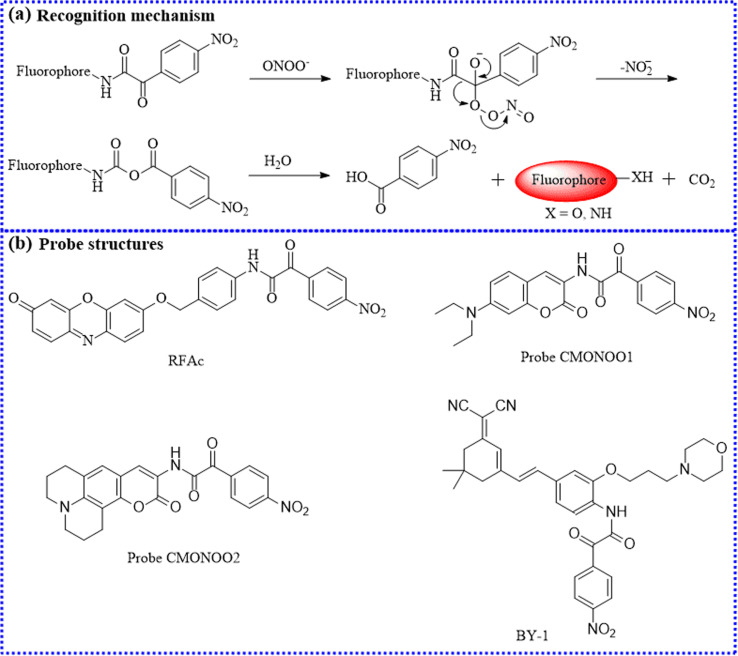
(a) The sensing mechanism between α-ketoamide and ONOO^−^. (b) The probe structures based on α-ketoamide.

A new resorufin core-based fluorescence probe (RFAc) was developed for detecting ONOO^−^ in living systems (Fig. 21).^102^ The α-ketoamide group in RFAc underwent nucleophilic addition and elimination reactions with ONOO^−^ to produce a free fluorophore with strong fluorescence. To our delight, RFAc exhibited high selectivity for ONOO^−^ without interference from other ROS species. With its good biocompatibility and low cytotoxicity, RFAc effectively tracked the intracellular ONOO^−^ levels. Encouraged by these promising results, RFAc was used for the imaging of endogenous ONOO^−^ fluctuations in the inflamed regions of an LPS-stimulated mouse model. Taken together, the authors clearly discovered that inflammatory sites emitted brighter fluorescence than the control sites, indicating that these regions generated abundant ONOO^−^.

The onset and progression of inflammation are often associated with elevated levels of ONOO^−^. Zhu's group utilized 3-aminocoumarin derivatives with a D-π-D configuration and 2-(4-nitrophenyl)-2-oxoacetamide to construct two small-molecule fluorescence probes (CMONOO1 and CMONOO2).^103^ The two probes both displayed fascinating detection performance, such as fast response time (roughly 60 s) and high selectivity. Subsequently, γ-carrageenan was selected as an inducer to produce inflammation in mice. Their findings uncovered a significant fluorescence increase in the inflamed regions over time, confirming that these sites continuously produced large amounts of ONOO^−^. As a result, these probes could be conducive to investigate the roles of ONOO^−^ in related diseases.

To examine lysosomal ONOO^−^ concentrations in osteoarthritis, an organic fluorescent probe (BY-1) with a large Stokes shift was reasonably proposed.^104^ Strikingly, the fluorescent intensity of BY-1 exhibited a strong linear relationship with ONOO^−^ concentrations. Satisfactorily, using BY-1, the actual levels of ONOO^−^ were monitored in living human chondrocytes cells under various experimental conditions. Moreover, BY-1 primarily accumulated in the lysosomes rather than other organelles due to the incorporation of a morpholine moiety. Importantly, BY-1 was utilized for the dynamic analysis of ONOO^−^ levels in a collagenase-stimulated osteoarthritis mouse model. Compared to other groups, the fluorescence values significantly increased in drug-induced osteoarthritis over time, indicating that osteoarthritis sites generated substantial amounts of ONOO^−^. Furthermore, BY-1 may serve as a diagnostic tool for evaluating the treatment efficacy of osteoarthritis.

#### 2.3.7 Fluorescent probes based on the indoline-2,3-dione group

**Fig. 22:**
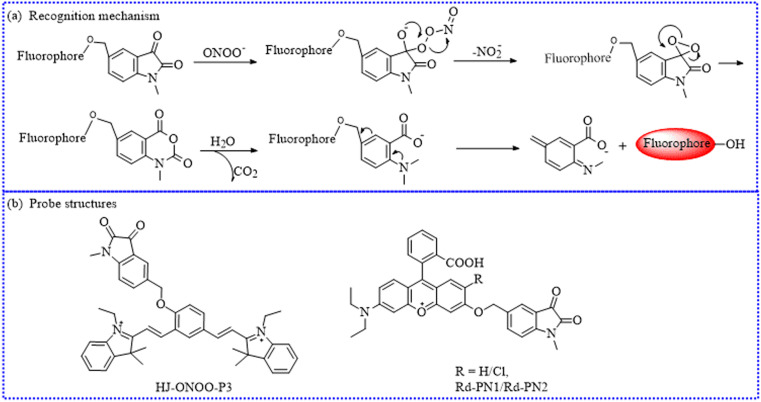
(a) The detection mechanism between indoline-2,3-dione and ONOO^−^. (b) The probe structures based on the indoline-2,3-dione group.

To explore the functionality of mitochondrial ONOO^−^, Li's group constructed a new NIR sensing probe (HJ-ONOO-P3) (Fig. 22).^105^ The interference of self-absorption could be effectively eliminated and the signal-to-noise ratio was significantly improved due to its substantial Stokes shift before and after reacting with ONOO^−^. Moreover, HJ-ONOO-P3 showed fast response (5 min) to ONOO^−^ in *in vitro* experiments. In addition, the indole moiety with a positive charge allowed HJ-ONOO-P3 to localize within the mitochondria. Moreover, this HJ-ONOO-P3 exhibited robust detection ability of intracellular ONOO^−^ levels under oxidative stress. Inspired by its effective monitoring abilities, HJ-ONOO-P3 was further utilized to monitor ONOO^−^ variations in living cells during oxygen–glucose deprivation/reperfusion and middle cerebral artery occlusion.

**Fig. 23:**
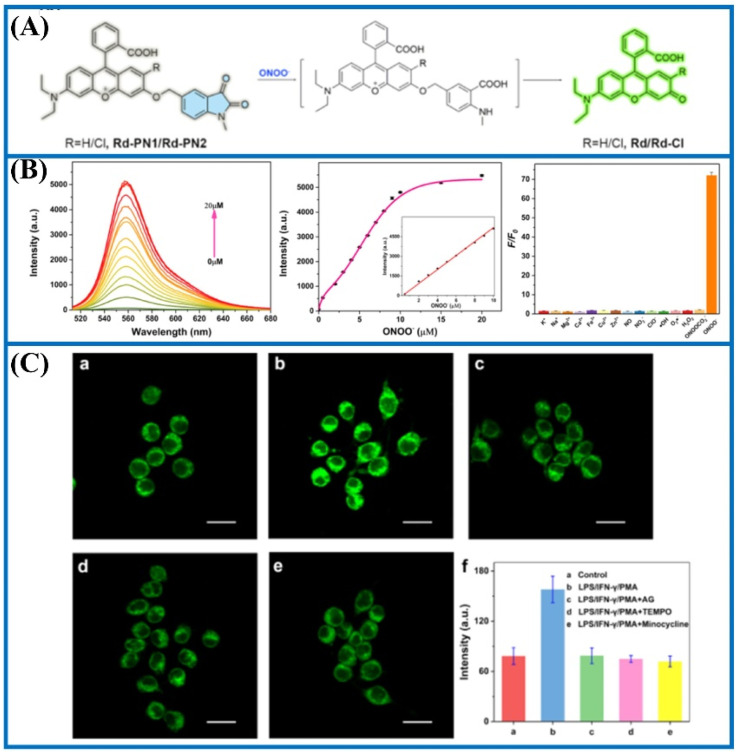
(A) The chemical structure of Rd-PN1/2 after adding ONOO^−^. (B) Fluorescence spectra of Rd-PN2 after the addition of ONOO^−^. (C) Confocal fluorescence images of Rd-PN2 in the presence of inducers in live cells. This figure has been reproduced from ref. 106 with permission from American Chemical Society, copyright 2020.

To uncover the underlying concern between ONOO^−^ and stroke, Liu's group constructed two small-molecule fluorescent sensors (Rd-PN1 and Rd-PN2) using an indoline-2,3-dione moiety as the triggering group and Rhodol derivatives as the ideal fluorophore.^106^ Compared to Rd-PN1, Rd-PN2, which contained a Cl group, showed more fascinating properties, including a large *F*/*F*_0_ value of approximately 71-fold. Moreover, Rd-PN2 showed a higher sensitivity to ONOO^−^ even in the presence of other ROS/RNS. Moreover, after Rd-PN2 reacted with ONOO^−^, the product showed a maximum two-photon active cross-section (152 GM). Additionally, cells emitted a strong fluorescent signal when stimulated with LPS, IFN-γ, and PMA (Fig. 23), indicating that a large amount of ONOO^−^ was expressed in these induced cells. However, with the addition of aminoguanidine (AG), 2,2,6,6-tetramethylpiperidinyl-1-oxide or minocycline, intracellular fluorescence was significantly inhibited owing to the elimination of ONOO^−^. Accordingly, using two-photon confocal fluorescence imaging, an increasing fluorescent signal was observed in brain microvessels in mice with ischemic stroke. These experiments demonstrated that excessive ONOO^−^ was produced during the rupture of cerebral microvessels. Consequently, these probes could serve as powerful tools for future studies on ONOO^−^-related diseases.

#### 2.3.8 Fluorescent probes based on the hydrazide group

**Fig. 24:**
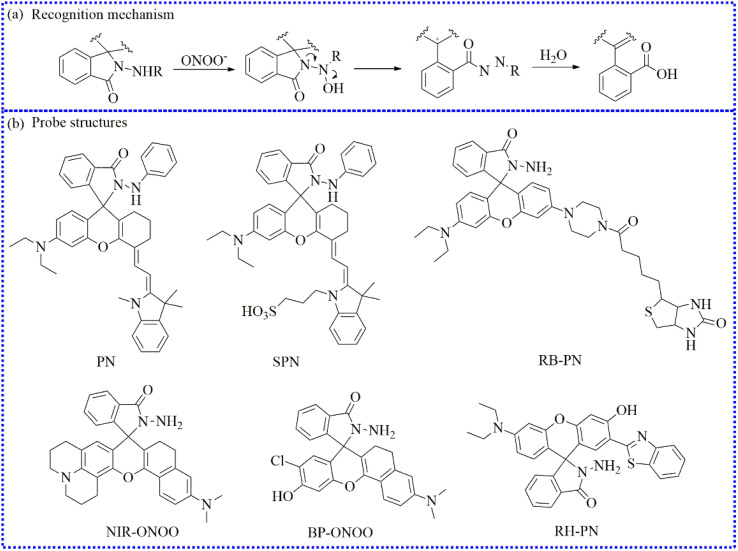
(a) The detection mechanism between hydrazide and ONOO^−^. (b) The probe structures based on the hydrazide group.

Hydrazide, a common recognition group for ONOO^−^, is extensively utilized in the preparation and application of molecular probes (Fig. 24).^121^ For instance, Xu's group synthesized an NIR fluorophore by incorporating indole and phenyl-hydrazine groups into rhodamine derivatives and then developed two organic NIR fluorescent probes (PN and SPN).^107^ Compared to PN, SPN, which was modified with an SO_3_ group on the branch chain, exhibited superior properties such as better water solubility, lower limit of detection, and good biocompatibility. Taken together, SPN enabled the specific investigation of endogenous and exogenous ONOO^−^ fluxes. Considering the characteristics of NIR excitation and emission, SPN was further tested for the super-resolution visualization of ONOO^−^ in zebrafish. APAP-incubated zebrafish exhibited brighter fluorescence than the control group. However, the fluorescence in stimulated zebrafish was significantly suppressed by NAC. The same phenomenon was clearly observed in mouse experiments. Meanwhile, these results proved that NAC had the ability to reduce the generation of ONOO^−^ in organisms and alleviate oxidative stress. Therefore, this study may facilitate the super-resolution imaging of ONOO^−^ in biological systems.

To detect ONOO^−^ levels in DILI, Zhang's group developed a two-photon excitation, NIR-emitting fluorescent sensor (NIR-ONOO) by expanding the conjugation system of rhodamine.^108^ The free probe was initially non-fluorescent due to its spirolactam structure. Upon reacting with ONOO^−^, the probe's structure generated a discernible fluorescence signal. NIR-ONOO exhibited several significant qualities toward ONOO^−^, mainly including a lower limit of detection (15 nM), good two-photon absorption cross section (54 GM), rapid response (10 s) and outstanding responsive enhancement (340-fold). Moreover, the imaging applicability of NIR-ONOO was explored in drug-induced hepatotoxicity. Notably, a gradual fluorescence enhancement was observed upon incubation with APAP, implying that these cells generated surplus ONOO^−^. Nevertheless, a decreased fluorescence signal was detected after preincubation with uric acid.

Jin's group optimized the Rhodol structure and prepared an ONOO^−^-activatable red-emitting fluorescent probe (BP-ONOO).^109^ Upon the addition of ONOO^−^, the hydrazide group in the probe underwent a ring-opening process. Among various species, BP-ONOO responded to ONOO^−^, requiring only 4 s. The fluorescence signals of the reacting solutions exhibited linearity with respect to the ONOO^−^ levels in calibration experiments. Notably, the excellent detection performance of BP-ONOO made it suitable for biological detection. Compared to the control cells, SIN-1-stimulated cells exhibited intense fluorescence. Moreover, these fluorescent signals progressively increased with the addition of SIN-1 doses. Additionally, the fluctuation in endogenous ONOO^−^ was imaged under stimulation with LPS and PMA.

To investigate the characteristics of ONOO^−^ in head and neck cancer, Huang's group developed a novel sodium-dependent multivitamin transporter (SMVT)-targetable fluorescent probe (RB-PN).^110^ The incorporation of the SMVT group enabled RB-PN to specifically bind to biotin, ensuring precise positioning. Using RB-PN, the ONOO^−^ influxes were successfully imaged in HNSCC cells. *In vivo* fluorescent imaging results confirmed that ONOO^−^ was overproduced in neck cancer.

Employing the ESIPT mechanism, Zhu's group synthesized a novel ratiometric fluorescent probe (RH-PN) by modifying rhodol with 2-(2′-hydroxyphenyl)benzothiazole.^111^ The resulting probe exhibited a distinct emission peak at 454 nm, which is attributed to 2-(2′-hydroxyphenyl)benzothiazole and the benzene ring. After pretreatment with ONOO^−^, the reacting solutions displayed a prominent absorption peak at 560 nm, accompanied by a color change from colorless to pink. Additionally, a distinct emission peak at 581 nm was observed in the buffer solution. Considering the ratiometric fluorescence intensity at these two wavelengths, the precision determination of ONOO^−^ was achieved. RH-PN demonstrated excellent monitoring capabilities for ONOO^−^, including a large Stokes shift (127 nm). Thus, the variations of ONOO^−^ fluxes in macrophage cells were directly visualized. Compared to the control cells, the ratiometric fluorescence value of the cells was decreased upon the addition of 4-amino-TEMPO. In contrast, the cells exhibited significant red fluorescence upon stimulation with ONOO^−^, PMA, and LPS. Subsequently, a similar phenomenon was observed in living zebrafish under the same experimental conditions.

#### 2.3.9 Fluorescent probes based on other reaction types

**Fig. 25:**
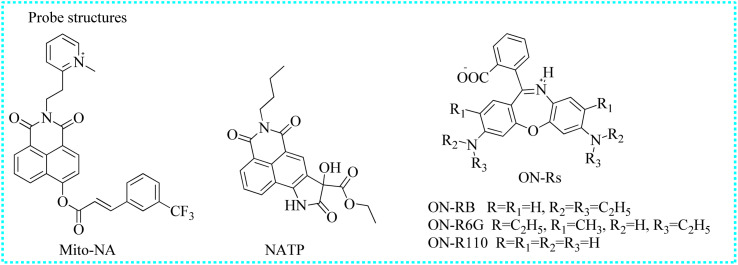
The probe structures based on other reaction groups.

Zhang's group established a novel ratiometric probe (Mito-NA) by regulating the ICT effect to monitor mitochondrial ONOO^−^ variations.^112^ The probe design incorporated a 1,8-naphthalimide framework decorated with 3-(trifluoromethyl) cinnamic acid and a pyridine cation (Fig. 25). Mito-NA exhibited short-wavelength fluorescence at approximately 450 nm due to the weak ICT mechanism. Upon interaction with ONOO^−^, the ester bond was broken and released the fluorophore with the hydroxyl group, resulting in a pronounced emission at 550 nm owing to the enhancement of the ICT process. This proportional detection allowed for the quantitative measurement of ONOO^−^. The introduction of pyridine cation enabled Mito-NA to aggregate in the mitochondria and monitor the changes in the ONOO^−^ content. Upon treatment with SIN-1, the cells exhibited strong fluorescence, resulting in a high ratio of green/blue channels in live cell lines (HeLa cells and RAW264.7 cells). Nevertheless, a low fluorescence intensity ratio (*F*_green_/*F*_blue_) was observed in uric acid-pretreated cells, indicating that Mito-NA could visualize mitochondrial ONOO^−^ fluctuations under different stimulations.

The levels of ONOO^−^ are closely associated with the initiation and progression of AD. Tang's team intelligently constructed a novel molecular probe (NATP) using a straightforward oxindole decyclization strategy.^113^ As expected, consistent with previous studies, ONOO^−^ interrupted the oxindole in the probe and then formed primary aniline derivatives, accompanied by a highly selective fluorescence enhancement response (within 10 s). Using NATP authors discovered that, amyloid-β triggered a significant amount of ONOO^−^ production and downregulated glutathione peroxidase 4 expression, causing ferroptosis in neurons. Additionally, the authors established a high-throughput screening platform based on a two-photon fluorescence probe to identify drug candidates with neuroprotective effects against amyloid-induced oxidative stress. More importantly, NATP possessed outstanding two-photon properties and blood–brain barrier penetration, enabling the *in situ* imaging of ONOO^−^ levels in the brain. This evidence contributed to clarifying the pathological effects related to ONOO^−^ in the progression of AD. Ultimately, this work provided a simple strategy for studying the potential relationships between ONOO^−^ concentrations and AD *in situ*.

Ma's group reported a panel of rhodamine derivatives featuring a variable π-conjugated design, which specifically reacted with ONOO^−^ to yield oxazines.^114^ These derivatives were easily synthesized through rearrangement under basic conditions. Single crystal data confirmed the presence of the 1,4-oxazepine heterocyclic core in the crystal structure. Meanwhile, these experimental results clearly determined the working mechanism of rhodamine derivatives and hydroxylamine. Among various ROS, only ONOO^−^-induced color changes in ON-RB, along with strong fluorescence in the NIR wavelength region. Most importantly, the generation and location of ONOO^−^ in macrophage-derived foam cells were recorded using ON-RB, envisioning that ONOO^−^ may be primarily produced in lipid droplets. Therefore, the chemical properties of rhodamine derivatives and their specific response to ONOO^−^ may advance the use of rhodamine derivatives for high-throughput bioimaging.

Researchers developed several small-molecule fluorescent probes for detecting and imaging ONOO^−^ in biological systems. Moreover, these fluorescent probes enabled the direct visualization of ONOO^−^ changes across different disease models, including AD, DILI, and tumor. Meanwhile, a number of ratiometric fluorescent probes accurately provided the exact relationship between ONOO^−^ changes and Golgi oxidative stress, such as MG-ONOO. Moreover, other probes showed striking imaging characteristics. For instance, the Rd-DPA3 not only ultrasensitively detected ONOO^−^ in *in vitro* tests (LOD = 3.4 nM) but also imaged the distribution of ONOO^−^ in the mouse brain with high spatio-temporal resolution. Interestingly, the ONOO^−^-responsive fluorescent probe (MBDP-Py^+^) was used to study the interaction of NAFLD with DILI. Furthermore, the fluorescence behaviors of MBDP-Py^+^ were conducive to understanding the mechanism and pathological process of NAFLD complicated with DILI. Particularly, the molecular mechanisms involving ONOO^−^ in the occurrence and development of AD were preliminarily elucidated using NATP. Additionally, the signaling pathways associated with the pathological processes of these reactive molecules had been mapped out to a certain extent. Therefore, the design of these molecular fluorescent probes offered a general construction strategy for the detection of other biomarkers.

## 3 Small-molecule fluorescent probes detect two of the three bioactive molecules (O_2_˙^−^, NO, and ONOO^−^)

**Fig. 26:**
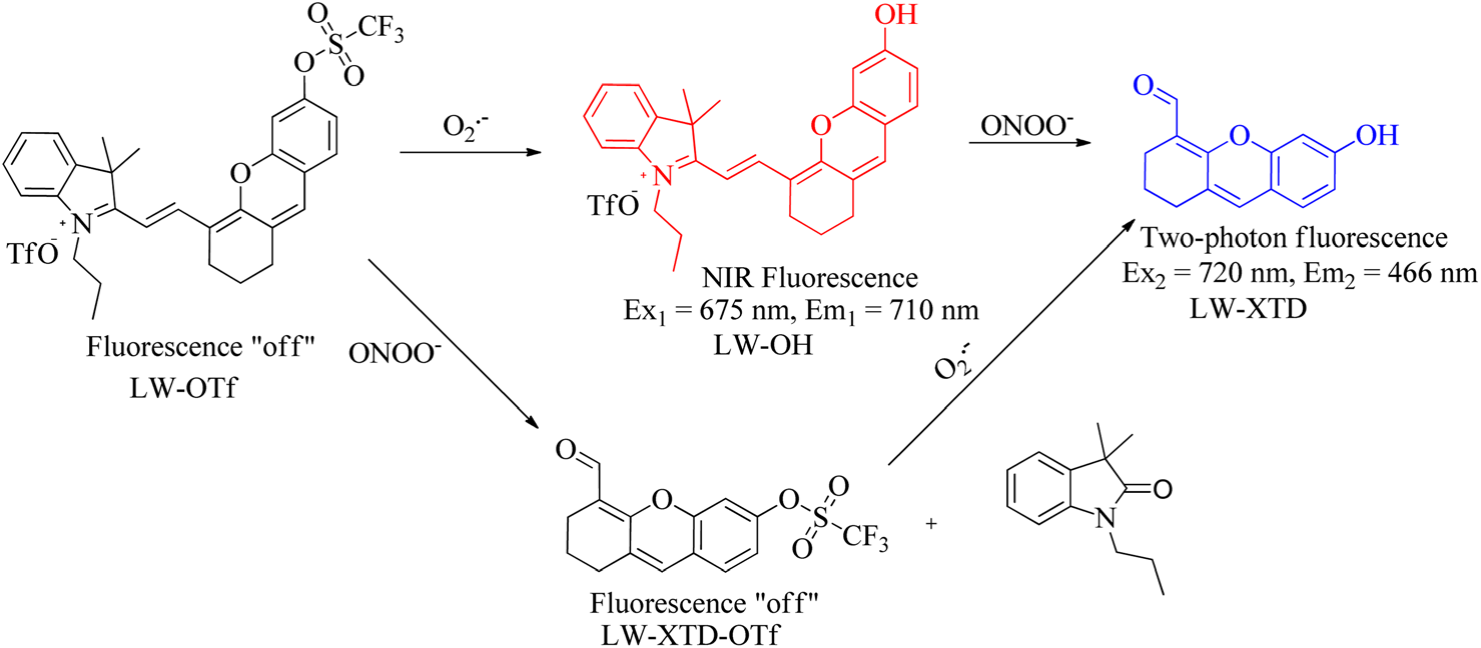
The probe structures and monitoring mechanism of the LW-OTf reaction with O_2_˙^−^ and ONOO^−^.

Understanding the relationship among the three reactive molecules (O_2_˙^−^, NO, and ONOO^−^) through the multi-component analysis of their changes in disease models is crucial in living systems. For example, previous reports showed that APAP-induced hepatotoxicity is closely associated with the overproduction of ROS/RNS.^122^ Tang's group successfully developed a reaction-type small molecule fluorescence probe (LW-OTf) with two-photon excitation and NIR emission properties (Fig. 26).^123^ Using LW-OTf, simultaneous detection of O_2_˙^−^ and ONOO^−^ was achieved during DILI. O_2_˙^−^ triggered the deprotection of the trifluoromethysulfonyl group, resulting in the release of free NIR fluorophore and enhancement of red fluorescence in *in vitro* experiments. Subsequent reaction with ONOO^−^ led to the oxidative cleavage of the C=C bond in the fluorophore, producing the oxanthene derivative with two-photon properties. LW-OTf was used as a diagnostic tool to monitor the chemoprotective effect of *tert*-butylhydroxyanisole in APAP-induced hepatotoxicity. Encouragingly, combining with hematoxylin and eosin staining confirmed the high influxes of O_2_˙^−^ and ONOO^−^ in the liver of DILI mice. As an ideal imaging and screening tool, LW-OTf could reveal molecular mechanisms related to drug-induced liver injury, early diagnosis and treatment, and efficacy evaluation.

## 4 Conclusion

We systematically reviewed recent advances in small-molecule fluorescent probes to detect O_2_˙^−^, NO, and ONOO^−^ in biological systems. We discussed in detail the construction tactics and working principles of some typical probes for these three reactive species. Fluorophores, including coumarin, 1,8-naphthalimide, BODIPY, semi-cyanine, and rhodamine, had been strategically paired with different reactive moieties to prepare some high-performing fluorescent probes. The luminescence process of these probes was closely related to various mechanisms, such as PET, ICT, and FRET. For example, the oxidation of the pyrocatechol unit was smoothly used for monitoring the dynamic and reversible fluctuation of O_2_˙^−^. Furthermore, NO reacted with the *o*-phenylenediamine unit, resulting in the cyclization and formation of a triazole structure, accompanied by a significant fluorescence change of probes owing to the inhibition of PET. Taken together, this rapid detection approach based on molecular fluorescent probes effectively monitored fluctuations in three bioactive molecules and revealed the underlying molecular mechanism.

Although the detection of O_2_˙^−^, NO and ONOO^−^ by organic fluorescent probes has been rapidly developed, there are still some shortcomings that need to be seriously considered in future studies. Firstly, these three reactive molecules are typically characterized by low concentrations, short lifetimes, and wide distributions. Future work should develop fluorescent probes with high specificity, sensitivity, rapid response times, and efficient localization capabilities. Among these factors, high specificity is particularly crucial because selectivity is an important indicator to measure a high-quality fluorescent probe. Although organic fluorescent probes have been developed, there are few new and specific recognition groups. According to previous works, a common strategy was to construct new fluorescent probes by linking recognition groups with different fluorophores, *i.e.*, some recognition groups were combined with different fluorophores to obtain probes with different properties. For example, the borate or boric acid structure could be used as a recognition group for ONOO^−^ or H_2_O_2_. *p*-aminophenol was used as a recognition group for ONOO^−^ or peroxidase. Orthophenylene and its derivatives served as the sensing unit for NO or carbonyl electrophiles (*e.g.*, methyl glyoyl). The trifluoromethanesulfonate and diphenyl phosphate groups could be used as recognition units for O_2_˙^−^ or ONOO^−^. Additionally, based on the existing structure and optical properties of small-molecule fluorescence probes, a series of fluorescence probes was obtained by adjusting the structure of the fluorophore. Afterwards, the properties of the obtained probes were tested in *in vitro* experiments and then the high-quality fluorescence probes were selected, including high selectivity and sensitivity. Notably, the selectivity of the targetable probe should be fully investigated in the presence of potential competing species in buffer solutions. To further validate the selectivity of the targetable probe in living cells, a specific scavenger or inhibitor can be incubated into the cells. Thus, researchers use specific inhibitors or scavengers to verify the selectivity of these probes in the cells. Besides, density functional theory can also optimize the structures of the proposed fluorescent probes. Secondly, the emission wavelength of these O_2_˙^−^-, NO-, and ONOO^−^-responsive small-molecule fluorescence probes is mainly concentrated in the short emission region, limiting their effectiveness for deep tissue imaging. Fortunately, the NIR-II region (1000–1700 nm) offers deeper penetration, lower background interference, reduced tissue damage, and super-resolution imaging. Developing O_2_˙^−^, NO, and ONOO^−^-responsive NIR-II fluorescent probes is promising for studying various disease processes. Thirdly, the optical probe with a single fluorescence signal is susceptible to microenvironment interference, such as excitation source and probe concentrations, resulting in inaccurate analysis results. Satisfactorily, the ratiometric small-molecule fluorescence probes have the capability of avoiding target-independent factors. Fourthly, dual-responsive small-molecule fluorescent probes with a single structure are rarely reported. Thus, dual-responsive probes should be developed for studying the mutual regulation and interactions. Noteworthily, developing molecular probes capable of simultaneously visualizing O_2_˙^−^, NO, and ONOO^−^ is critical for understanding and revealing the physiological, biochemical, and pathological functions in biological systems. Finally, some of the underlying mechanisms have been revealed using these fluorescent probes. Hence, follow-up work can further explore the pathogenesis of diseases with the help of existing probes and other technologies. In conclusion, this review is expected to provide valuable insights into the future development of small-molecule fluorescent probes for the detection of O_2_˙^−^, NO and ONOO^−^ in complex systems.

## Data availability

No data was used for the research described in the article.

## Author contributions

Yongqing Zhou, Shan-Shan Zhang, Mei Yan and Juyoung Yoon made substantial contributions to discussions of the content. Yongqing Zhou prepared the draft manuscript. Xuan Kuang, Xiaofeng Yang, Juan Li and Xianzhe Wei helped to prepare the draft manuscript. Won Jun Jang, Shan-Shan Zhang, Mei Yan and Juyoung Yoon revised the manuscript before submission.

## Conflicts of interest

The authors declare that they have no known competing financial interests or personal relationships that could have appeared to influence the work reported in this paper.
